# Feasibility and reliability of sequential logic with gene regulatory networks

**DOI:** 10.1371/journal.pone.0249234

**Published:** 2021-03-30

**Authors:** Morgan Madec, Elise Rosati, Christophe Lallement

**Affiliations:** Laboratory of Engineering Sciences, Computer Sciences and Imaging, UMR 7357 (University of Strasbourg / CNRS), Illkirch, France; Universitat Pompeu Fabra, SPAIN

## Abstract

Gene regulatory networks exhibiting Boolean behaviour, e.g. AND, OR or XOR, have been routinely designed for years. However, achieving more sophisticated functions, such as control or computation, usually requires sequential circuits or so-called state machines. For such a circuit, outputs depend both on inputs and the current state of the system. Although it is still possible to design such circuits by analogy with digital electronics, some particularities of biology make the task trickier. The impact of two of them, namely the stochasticity of biological processes and the inhomogeneity in the response of regulation mechanisms, are assessed in this paper. Numerical simulations performed in two use cases point out high risks of malfunctions even for designed GRNs functional from a theoretical point of view. Several solutions to improve reliability of such systems are also discussed.

## Introduction

Simple boolean functions have been routinely implemented with gene regulatory networks (GRNs) for the last two decades [1–10]. For most of them, the output signal depends only on the current input signal. Another way of saying this is that the output value is unique for each input combination. Because of this property, such circuits are called “combinatorial” in electronics and can be simply described by a truth table [11]. However, achieving more sophisticated functions often requires advanced digital functions such as memories, counters or automata, whose behaviour depends not only on the current value of input signals but also on their previous ones. An alternative way of describing it is to say that the output signal depends both on the current state of the system and its inputs. In digital electronics, systems with that kind of behaviour are called “sequential” systems, or “finite state machines”. The implementation of such systems in biology would enable a GRN to react on a timed sequence of stimuli rather than on a single event (*e*.*g*. counting the number of stimuli, having a different behaviour depending on which stimulus comes first, generating a sequence of outputs controlled by one or multiple inputs, etc.).

Sequential GRNs are not so commonplace in the literature. Besides the well-known toggle switch [1], most of them are alternative memory elements such as JK-latches [12, 13] or toggle memories [14]. More recently, Andrews *et al*. made the proof of concept of state machines controlled by checkpoints with an architecture based on a cascade of toggle switches [15]. In 2014, Oishi and Klavins described a theoretical framework for the engineering of any finite state machine with GRNs using a small set of promoters and transcription factors [16]. This approach has been validated with numerical simulations only. Along the same line, more complex systems, such as conditional memories [17], synchronous memories [18, 19], counters [20, 21], finite-state automata [22] and control units [23] have also been designed *in silico* but without actual experimental implementation. In this paper, the focus is put on GRN-based synthetic biology, although this is not the only way to build artificial biological systems with sequential functions. Alternatives such as protein-protein interactions [24], DNA origami [25] or post-transcriptional regulations [26, 27] have already been demonstrated.

The construction of sequential functions with GRNs always requires positive feedback loops. These loops introduce multi-stability, which is required to store the current state of the system, and also define the concept of internal variables (*i*.*e*. signals that are neither inputs nor outputs but that encodes the current state of the system). A sequential GRN can always be decomposed into two subunits. On the one hand, the output logic, or output GRN, computes outputs of the system as a function of inputs and the current state. On the other hand, the transition logic, or transition GRN, computes the next state of the system as a function of inputs and the current state.

**Fig 1:**
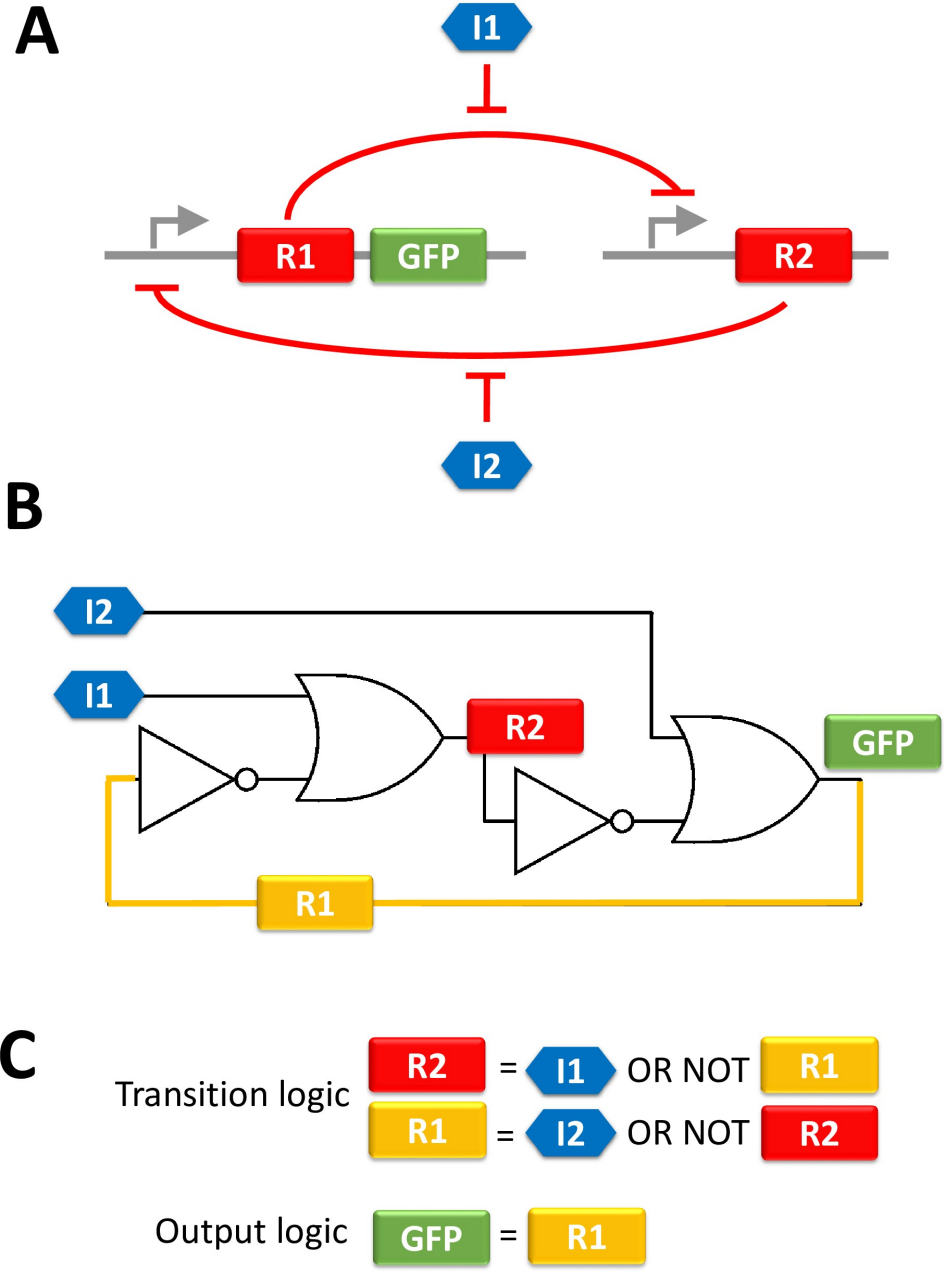
Three representations of Gardner’s toggle switch. (A) corresponds to the biological sketch of the GRN. (B) is the equivalent electronic circuit. The orange wire corresponds to the internal variable R1. (C) is the Boolean equations corresponding to this system. We can see that the production of R1 by itself, forming a positive feedback loop: the production of R1 is negatively regulated by R2 which production is in turn negatively regulated by R1. This is always the case for internal variables. It should also be noticed that, as the system is symmetric, an alternative way of interpreting this regulation is to consider R2 as the internal variable.

Properties of sequential GRNs can be illustrated on the aforementioned toggle switch [1]. The biological sketch of this system is described in Fig 1A. It consists of two constructs, each of them being composed of a promoter and a coding sequence for a transcription factor that negatively regulates the promoter of the other construct. The system has two stable steady states: one for which the left gene is active (*i*.*e*. R1 is produced) and another for which the right gene is active (*i*.*e*. R2 is produced). The switch from one state to the other is triggered by the release of the negative regulation by an external stimulus (I1 or I2). When no external stimulation is applied, the system remains in its current state. Thus, it is a sequential system. The electronic equivalent circuit of this GRN is drawn in Fig 1B. The feedback (in orange) and the internal signal R1 are identified. Boolean equations corresponding to the system are given in Fig 1C.

Mathematically, a sequential system is described by a state diagram composed of states and transitions between states. Its design consists of finding the Boolean equations for both the output logic and the transition logic. Several algorithms exist to perform this task, one of the most common being Huffman-Mealy’s method [11]. Once the Boolean equations are established, a genetic design automation tool [28–30] can be applied to get the corresponding GRN.

As described above, one feature of sequential systems is the existence of positive feedback loops. Unfortunately, such loops also tend to increase the risk of instability and malfunction. Issues of this kind are known under the generic term of “critical races” [11]. They may also happen in electronics, despite the high reliability of the static and dynamic response of digital circuits, but are even more likely to occur in biology where the response of a digital gate may change from one couple promoter / transcription factors to the other [15].

This paper aims at evaluating the risk of critical races in sequential GRNs, and more generally speaking, assessing the feasibility and the reliability of such systems. The discussion relies on numerical simulations performed on two sequential GRNs.

## Material and methods

### Design of the system A

**Fig 2:**
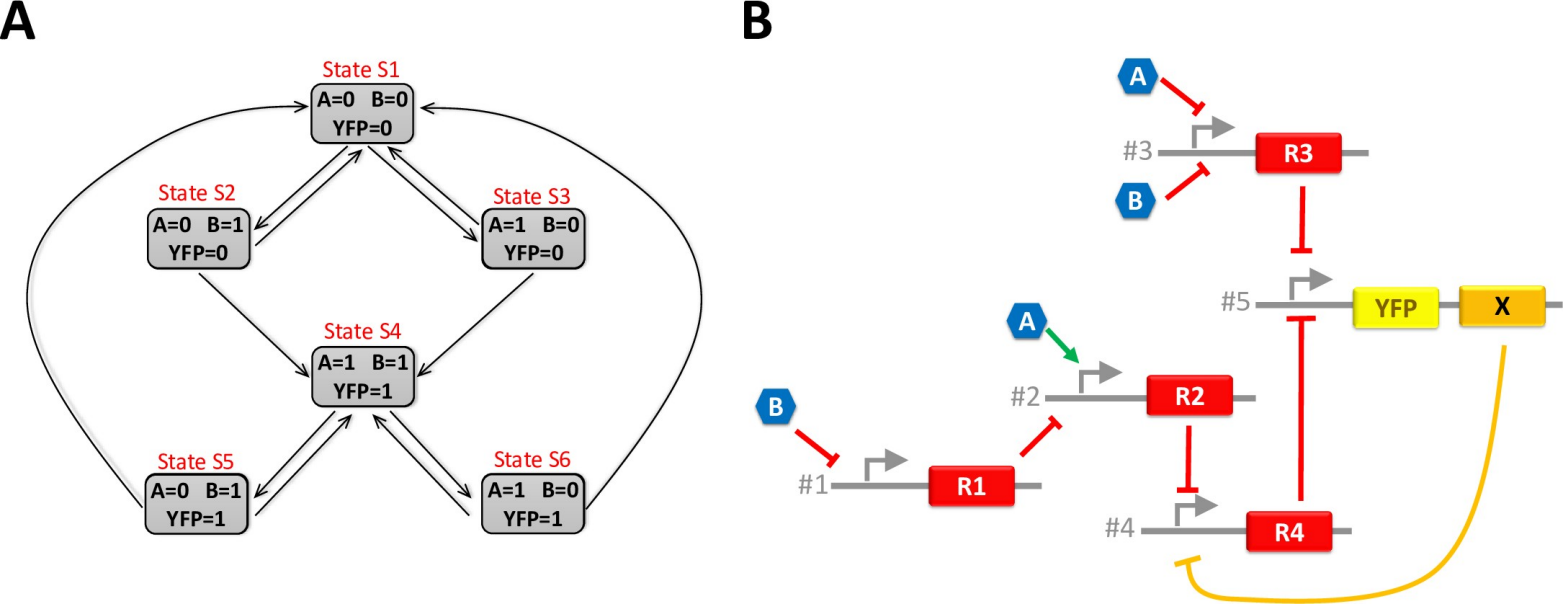
State diagram and GRN associated with system A. (A) is the state diagram of system A. (B) is a GRN that meets the requirement of system A. The orange path identifies the positive feedback loop, which introduces bistability in the GRN: X inhibits the production of R4 which itself inhibits the production of X.

System A operates as follows: “*The system is composed of two inputs (A and B) and one output (YFP)*. *The output only goes high if both inputs are high*. *Conversely*, *the output goes low if both inputs are low*. *Otherwise*, *the output stays at its previous state*.*”* This behaviour can be summarised by the 6-state diagram of Fig 2A. In such a representation, each state corresponds to a given combination of inputs and outputs and transitions between states occur along with the arrows when the combination of inputs changes. For instance, let us first consider that the system is in the state *S*1 and that both inputs are low. In this state, *YFP* is also low. If *A* rises, the system goes into the state *S*3 but the *YFP* remains low. If *A* returns to low, the system goes back to state *S*1. Otherwise, if *B* also rises, the system goes in state *S*4 and *YFP* also rises. The state diagram shows that several states share the same input combination but produce a different output (*e*.*g*. *S*2 and *S*5 on the one hand and *S*3 and *S*6 on the other hand). This is evidence that the system is sequential and, thus, cannot be achieved without positive feedback.

Huffman-Mealy’s method is then applied to calculate the Boolean equations ruling the system from its state diagram [11]. The method first establishes the minimal number of internal variables required (one in our case). Second, states are encoded with this internal variable called *X*. The state encoding is arbitrary. Let us choose for instance *X* = 0 for states *S*1, *S*2 and *S*3 and *X* = 1 for states *S*4, *S*5, and *S*6. Third, the truth tables giving the next value of the internal variable (*X*’) and the output (*YFP*) as a function of system inputs (*A* and *B*) and the current value of the internal variable (*X*) are established. Fourth, these truth tables are then solved to obtain the corresponding Boolean equations:

$$X^{\prime }=A∙B+X∙(A+B)$$ (1)

YFP=A∙B+X∙(A+B) (2)

for the internal variable and for the output. The Huffman-Mealy’s method and the design process of the System A are detailed in (S1 File).

### Design of the system B

System B operates as follows: “*the system is composed of two inputs (A and B) and one output (YFP)*. *The system reacts on any set of two consecutive pulses on A and/or B*. *The output YFP rises at the beginning of the first pulse and falls at the end of the second pulse*. *Four possible scenarios have to be considered*: *i) both pulses are performed with the same input*, *ii) pulses are performed with different inputs but the second pulse starts after the end of the first one*, *iii) pulses are performed with different inputs and the second pulse starts before the end of the first one and iv) pulses are performed with different inputs and the second pulse takes place entirely during the first one*.*”*

**Fig 3:**
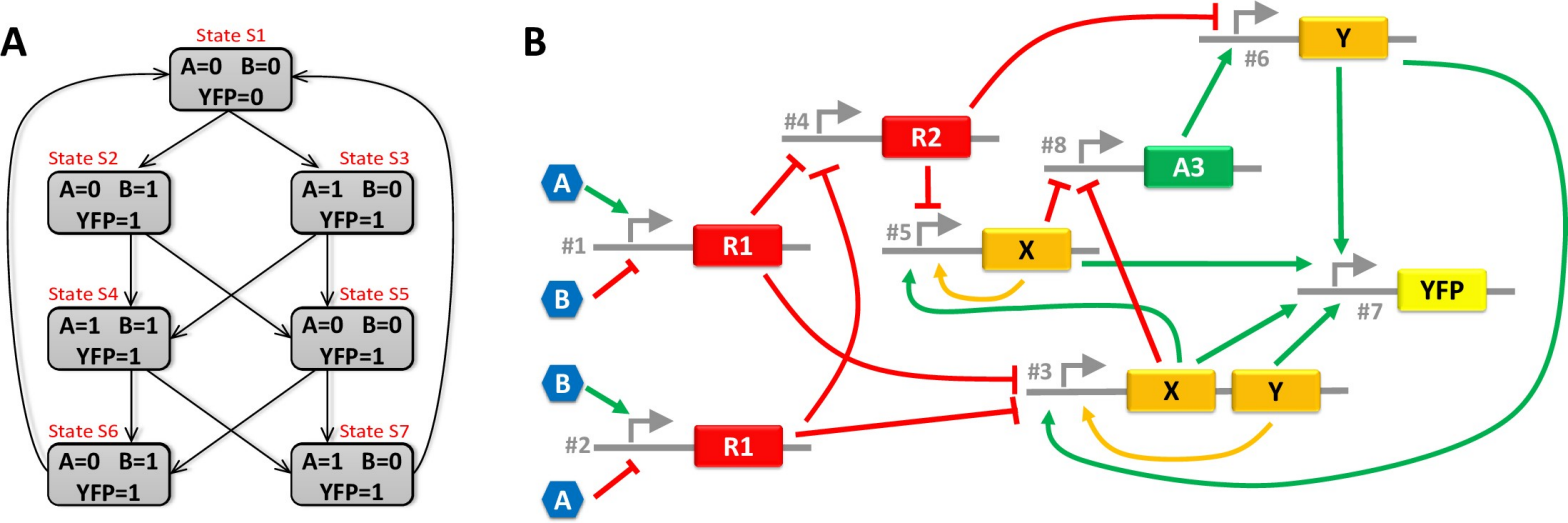
State diagram and GRN associated with system B. (A) is the state diagram of system B. (B) is a GRN that meets the requirement of System B. The orange paths identify the positive feedback loops, which are straightforward in this GRN.

The 7-state diagram of Fig 3A summarises the behaviour of system B. Huffman-Mealy’s method is applied again to obtain the Boolean equations ruling the system from this state diagram. This time, the minimal number of internal variables is two. Let *X* and *Y* be these internal variables. The equations giving the next combination of internal variables (*X*’ and *Y*’) and the output (*YFP*) as a function of the input (*A* and *B*) and the current combination of internal variable (*X* and *Y*) are:

$$X^{\prime }=Y∙(\bar{A}∙\bar{B}+A∙B)+X∙(\bar{A}∙B+A∙\bar{B}),$$ (3)

$$Y^{\prime }=Y∙(\bar{A}∙\bar{B}+A∙B)+\bar{X}∙(\bar{A}∙B+A∙\bar{B})$$ (4)

for internal variables and

YFP=A+B+Y (5)

for the output. With more than one internal variable, the state encoding is not unique and the same state diagram may lead to completely different GRN. Some choices appear more clever than others and lead to simpler and more reliable GRN. To illustrate that purpose, an alternative state encoding is considered. It leads to the following equations for the internal variables (equation for the output is the same):

$$X^{\prime }=\bar{A}∙\bar{B}∙\bar{X}∙Y+X∙(A+B)+A∙B+X∙\bar{Y},$$ (6)

$$Y^{\prime }=\bar{A}∙B+A∙\bar{B}.$$ (7)

The design of the System B is detailed in (S2 File).

### GRN construction

The construction of GRNs from the Boolean equations, *i*.*e*. Eqs (1)–(7), is performed with GeNeDA, a genetic design automation tool derived from the field of digital electronics [31]. GeNeDA computes the optimal GRN that matches a Boolean equation by assembling abstracted biological parts (promoters / transcription factors couples) from a library. In our case, the library is composed of four abstracted parts:

- A promoter with a single repressor (*e*.*g*. promoter #1 of the GRN on Fig 2B)
- A promoter with one activator and one repressor (*e*.*g*. promoter #2 of the GRN on Fig 2B).
- A promoter with two repressors (*e*.*g*. promoter #3 of the GRN on Fig 2B).
- A promoter with two activators (*e*.*g*. promoter #3 of the GRN on Fig 3B).

Examples of actual biological constructs corresponding to these abstracted parts can be found in the literature for several types of bacteria. For example, ten different promoters with a single repressor have been demonstrated in [15] for *E*. *coli*. In [10], three constructs of a promoter with two repressors are shown. They are combined together to perform an XOR function. Examples of promoters with two activators are also given in the same paper. Other examples of constructs in *E*.*Coli* can be found in [32]: J. Shin *et al*. tested eighteen different NOT and NOR gates, *i*.*e*. promoter with one or two repressors. Their GRNs are also composed of promoters with one activator and one repressor. However, the activation is sometimes rather a release of repression on the promoter than a true activation of the promoter. Finally, two activators on a single promoter can also be replaced by a single activator synthesised by different constructs to perform an OR function. This strategy is used for R1, X and Y in Fig 3B. This literature review shows that, on the one hand, there is a wide variety of constructs performing the elementary functions assembled by the synthesiser and that, on the other hand, the assembly of several of these constructs to obtain complex logical functions has already been demonstrated, notably in [32].

The GRN designed by GeNeDA for both systems are respectively sketched in Figs 2B and 3B. Five promoters and five transcription factors (one of them being the internal variable) are required for the system A. Eight promoters and six transcription factors (two of them being the internal variables) are required for the system B. For the sake of simplicity, we consider that a molecule can play both the role of an activator for one promoter and a repressor for the other (*e*.*g*. X of the GRN of Fig 3B). In practice, I might be necessary to use two different molecules with coding sequence associated with the same promoter. Thus, they can be considered as the same molecule, at least from the modelling perspective. For both systems, a possible correspondence between abstracted transcription factors (R1, R2, etc.) and actual transcription factors characterised by Shin et al. (amtR, lmrA, etc.) is given in for both systems (S1 File for System A and S2 File for System B).

### Boolean model and event-driven simulation

The behaviour of the system at the Boolean level is described in VHDL, a hardware description language dedicated to the modelling and the simulation of digital electronic circuits [33]. VHDL enables the modelling of a system at different levels of abstraction. In our case, three different models are written for both systems:

- The behavioural model corresponds to a direct and procedural translation of the state diagram in VHDL. Thus, it can be considered as a reference.
- The ideal model of the GRN corresponds to the VHDL transcription of equations given by Huffman-Mealy’s method and the GRN synthesis. Thus, it can be used to validate the GRN design procedure.
- The delayed model for the GRN uses the same equations as the ideal model except that each regulation process introduces a delay in the circuit. These delays model actual delays of the biological processes (*e*.*g*. time between the start of the production of an mRNA and the start of the production of the molecule it encodes).

All models are simulated with a commonplace event-driven simulator, namely ModelSim from Mentor Graphics [34]. The input stimuli are fixed to cover all possible state transitions.

### Dynamic models

Dynamic models are automatically generated from Boolean equations and written in MATLAB. Models are composed of a set of ordinary differential equations (ODEs), one per involved transcription factor and one per associated mRNA. Regulations are introduced as a modulation of the transcription rate according to Hill’s equation [35]:

$$k_{TR,eff}(\left[A],[R\right])=k_{TR,max}∙(\alpha +\frac{1-\alpha }{\left({1+(\frac{K_{A}}{\left[A\right]})}^{n_{A}})∙(1+{\left(\frac{\left[R\right]}{K_{R}}\right)}^{n_{R}}\right)}).$$ (8)

Thus, the ODE are

$$\frac{d[mRNA_{i}]}{dt}=k_{TR,eff}(\left[A],[R\right])-d_{mRNA_{i}}∙[mRNA_{i}]$$ (9)

for the transcription process and

$$\frac{d[P_{i}]}{dt}=k_{TL,eff}∙[mRNA_{i}]-d_{i}∙[P_{i}]$$ (10)

for the translation process. Quantities and parameters involved in Eqs (8)–(10) are listed in Table 1. For all molecules, concentrations are normalised such as the concentration of the protein (or mRNA) inside the cell is bounded between 0 and 1. An effective translation rate *k*_*TL*,*eff*_ and transcription rate *k*_*TR*,*eff*_ are used in Eqs (9) and (10) for that purpose. The default values for dynamic parameters have been fixed according to standard values given in [36] for *E*.*Coli*. Other parameters are set arbitrarily to have a fold ratio of 1000 between the active and inactive states. Finally *K*_*A*_ and *K*_*R*_ are adjusted to the middle of the dynamic range of the concentration of the transcription factors in log scale. The set of ODEs is numerically integrated with MATLAB. The solver is an explicit fourth-order Runge-Kutta algorithm (*ode45* function in MATLAB) [37].

**Table 1:** Default parameters used for the dynamic model of the GRN.

Symbol	Description	Default value
[*A*]	Normalised concentration of activator	-
[*R*]	Normalised concentration of repressor	-
[*mRNA*_*i*_]	Normalised concentration of mRNA encoding for the molecule *P*_*i*_	-
[*P*_*i*_]	Concentration of the synthesised molecule *P*_*i*_	-
*k*_*TR*,*eff*_	Maximal effective transcription rate (to obtain normalised conc.)	0.002 s^-1^
*α*	Promoter leakiness	0.001
*K*_*A*_	Dissociation constant of the activator with its binding site	0.03
*K*_*R*_	Dissociation constant of the repressor with its binding site	0.03
*n*_*A*_	Hill’s number for the activator	2
*n*_*R*_	Hill’s number for the repressor	2
*k*_*TL*,*eff*_	Effective translation rate (to obtain normalised concentrations)	10^−3^ s^-1^
$d_{mRNA_{i}}$	Degradation rate for the mRNA encoding for the protein *P*_*i*_	0.002 s^-1^
*d*_*eff*,*i*_	Degradation rate for the protein *P*_*i*_	10^−3^ s^-1^

### The critical race issue

In digital electronics, a “critical race” is a generic term that describes a malfunction that occurs in a sequential system due to a lack of synchronisation during state transitions. Most of them are caused by uncontrolled propagation delays of logic gates involved in the feedback path. Consequences of a critical race can be observed in the state diagram of system B (Fig 3A). This system has two internal variables, *X* and *Y*. Let us consider that the state *S*1 is encoded with *X* = 0 and *Y* = 0, the state *S*2 and *S*3 with *X* = 0 and *Y* = 1, the state *S*4 and *S*5 with *X* = 1 and *Y* = 0 and the state *S*6 and *S*7 with *X* = 1 and *Y* = 1. Assume that we are in the state *S*6. In this state, *A* = 0, *B* = 1, *X* = 1 and *Y* = 1. When *B* falls a transition to the state *S*1 is planned. This implies a simultaneous switch of both internal variables. In practice, if *Y* switches before *X*, the system goes through a transient state in which *A* = 0, *B* = 0, *X* = 1 and *Y* = 0. Thus, the system believes it is in the stable state *S*5. As a consequence, the system will stay in the state *S*5, instead of reaching *S*1.

### Simulation of the open-loop GRN

The open-loop GRN corresponds to the GRN without any feedback. For instance, the open-loop GRN of the system A is the one described in Fig 2B without orange regulation. When feedback is broken, X becomes an external input (as for A and B). To perform simulations of the complete GRN and the open-loop GRN in the same conditions, specific stimulus, which corresponds to the expected timing diagram provided by the event-driven simulation of the complete GRN, has to be applied to X. The same approach is used for system B. Sketches of the open-loop GRN as well as the timing diagram used for its simulation are given in (S1 File for system A and S2 File for System B).

The simulation of the open-loop GRN, in parallel with the complete GRN is of interest in case of malfunctions; it helps to discriminate whether the malfunction comes from a bad computation of the feedback or from a bad synchronisation when feedback is applied.

### Impact of the parameters inhomogeneity

In this subsection, the methodology used to assess the sensibility of systems towards the dispersion of the regulation parameters is described. The dynamic model of each GRN depends on a large number of parameters: transcription rates, translation rates, degradation rates for mRNA and transcription factors, regulation parameters. Our study focuses on the two that might exhibit the higher variability from one regulation to another [15], namely the dissociation constants of the transcription factor with its binding site (*K*_*A*_ for activators and *K*_*R*_ for repressors) and Hill’s number (*n*_*A*_ for activators and *n*_*R*_ for repressors). Let Λ^(0)^ be the vector gathering the default value of these parameters for a given GRN. For example, in the case of System A, we have

$$\Lambda^{\left(0\right)}=\left[\begin{array}{c} K_{R,B\to 1},K_{R,R1\to 2},K_{A,A\to 2},{K_{R,A\to 3},K_{R,B\to 3},K}_{R,R2\to 4},K_{R,X\to 4},K_{R,R3\to 5},K_{R,R4\to 5},\\ n_{R,B\to 1},n_{R,R1\to 2},n_{A,A\to 2},{n_{R,A\to 3},n_{R,B\to 3},n}_{R,R2\to 4},n_{R,X\to 4},n_{R,R3\to 5},n_{R,R4\to 5} \end{array}\right]$$ (11)

where *K*_*R*,*B*→1_ and *n*_*R*,*B*→1_ are, for instance, the regulation parameters of the transcription factor B on the promoter of the construct #1, *K*_*R*,*R*1→2_ and *n*_*R*,*R*1→2_ the regulation parameters for the repressor R1 on the promoter of the construct #2, etc.

For a given inhomogeneity value *σ*, a test is composed of 100 runs with 100 different parameters sets (Λ^(1)^,…,Λ^(100)^) generated by adding a random variable to each element of Λ^(0)^. The random variable is applied in the linear domain for Hill’s number and the logarithmic domain for the dissociation constant.

$$n_{A/R,x}^{\left(i\right)}=n_{A/R,x}^{\left(0\right)}∙\Psi (1,\sigma )$$ (12)

and

$$K_{A/R,x}^{\left(i\right)}=10^{\log_{10}\left(K_{A/R,x}^{\left(0\right)})∙\Psi (1,\sigma \right)}.$$ (13)

with $n_{A/R,x}^{\left(i\right)}$ and $K_{A/R,x}^{\left(i\right)}$ the parameters associated with the regulation *x* in the vector Λ^(*i*)^, *σ* the inhomogeneity value and Ψ(*μ*, *σ*) a random variable drawn from a Gaussian distribution centred on *μ* and with a standard deviation of *σ*. It should also be noted that Hill’s number always have to be positive. Thus, parameter sets with at least one negative $n_{A/R,x}^{\left(i\right)}$ are skipped and replaced. Examples of distributions and ranges for the values of $n_{A/R,x}^{\left(i\right)}$ and $K_{A/R,x}^{\left(i\right)}$ as a function of *σ* are given in (S3 File).

Dynamic simulations are then performed for each Λ^(*i*)^ and the simulation result is compared to the expected response (*i*.*e*. the response provided by the event-driven simulation with the same input stimuli). For that purpose, the simulation result of the dynamic model is sampled and binarised. The sampling is performed just before each event (change of inputs) in order to give the system as much time as possible to reach the steady state. The binarisation is performed by comparison with a threshold fixed to the middle of the dynamic range of the concentrations in logarithmic scale, *i*.*e*. 0.03. A run is considered to be successful is the sampled and binarised response matches with the expected response. For a given *σ*, the metric used to evaluate the impact of inhomogeneity is the percentage of Λ^(*i*)^ Leading to a successful run.

### Impact of stochasticity of biological processes

In this subsection, the methodology used to assess the robustness of GRNs towards the biological noise is described. Biological noise is mostly due to stochasticity in gene expression [38]. The gold standard for the simulation of stochasticity in biological processes is Gillespie’s algorithm [39]. However, this approach is very greedy from a computation time perspective, especially in our case where systems are large and where the analysis required a large number of simulations. An alternative to Gillespie’s algorithm consists of adding a random variable, or biological noise, at each time step during the deterministic simulation [40]. It is less accurate but lighter in terms of computation power. Previous works already shown that such an approach leads to comparable results as Gillespie’s approaches as soon as the number of occurrences of each molecule is high enough, which is our assumption for this study [40, 41].

In practice, each term of the deterministic dynamic model, *i*.*e*. the right terms of Eqs (9) and (10), is multiplied by a random variable:

$$\frac{d[mRNA_{i}]}{dt}=k_{TR,eff}(\left[A],[R\right])∙\psi_{m_{i}}(t)-d_{mRNA_{i}}∙[mRNA_{i}]∙\psi_{{dm}_{i}}(t)$$ (14)

$$\frac{d[P_{i}]}{dt}=k_{TL,eff}∙[mRNA_{i}]∙\psi_{i}(t)-d_{i}∙[P_{i}]∙\psi_{d_{i}}(t).$$ (15)

$\psi_{m_{i}}(t)$, $\psi_{{dm}_{i}}(t)$, *ψ*_*i*_(*t*) and $\psi_{d_{i}}(t)$ are random variables drawn from normal distribution centred on 1 and with a standard deviation of *σ*. The parameter *σ* is called the “noise level” in the following. If the biological mechanisms are true Poissonian processes, the theoretical value of *σ* is 1. However, this analysis is carried out with values for *σ* from 0.01 to 10 in order to have a better assessment of the impact of the noise.

Again, for each value of *σ*, a test is composed of 100 runs. The success of a run is evaluated in the same way as for the parameters inhomogeneity except that the sampling is performed by averaging over a 100-point window before each event rather than on a single point.

## Results

### Simulation at the Boolean level

**Fig 4:**
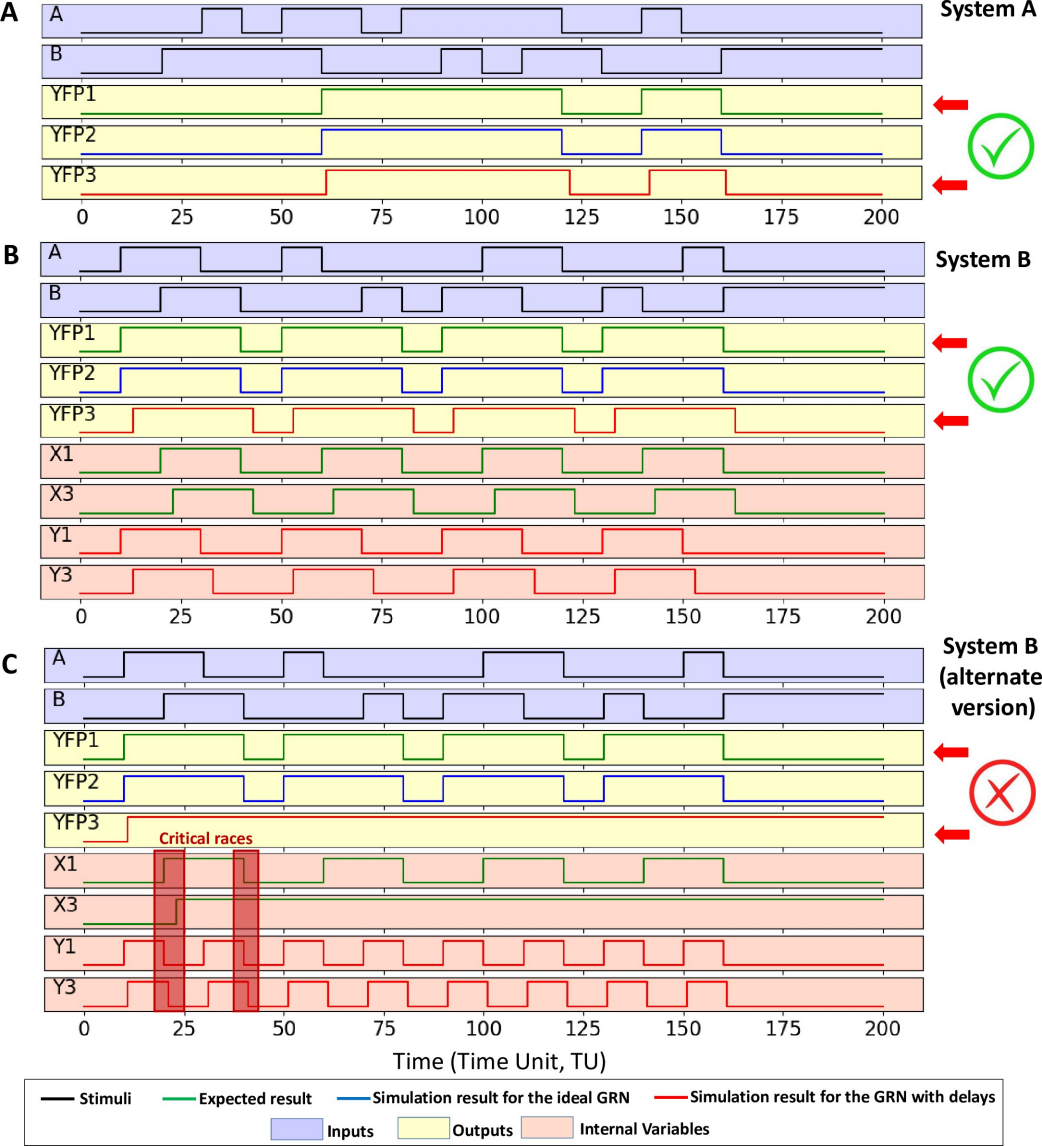
Simulation results for the Boolean models obtained with VHDL models simulated with ModelSim. (A) corresponds to the simulation results for system A. (B) and (C) corresponds to simulation results for both versions of system B. For both systems, green curves are obtained with the behavioural model of the system (YFP1, X1 and Y1). Blue curves are obtained with the ideal model of the GRN (YFP2). Red curves are obtained with the delayed model of the GRN (YFP3, X3 and Y3). Curves with a blue background corresponds to the inputs of the system (A and B). Curves with a yellow background corresponds to the system’s output (YFP*n* with *n* depending on the model used). Curves with an orange background corresponds to the internal variables (X*n* and Y*n*, with *n* depending on the model used). The scale of x-axis is an arbitrary time unit (TU). Input events occur every 10 TUs and the elementary delay introduced for each regulation mechanism in the delayed model is fixed to 1 TU. In (C), critical race issues are highlighted at 20 and 40 TU.

The simulation results of both systems at the Boolean level are given in Fig 4 for the three models described in the “*Boolean Model and Event-Driven Simulations*”Section of Materiel and Methods. In the following discussion, let *YFP*1, *X*1 and *Y*1 be respectively the output and the internal variables waveforms obtained with the behavioural model, *YFP*2, *X*2 and *Y*2 respectively the output and the internal variables waveforms obtained with the ideal model and *YFP*3, *X*3 and *Y*3 respectively the output and the internal variables waveforms obtained with the delayed model.

From Fig 4A, it can be pointed out that designed GRN for the system A works properly: the waveform of *YFP*1 and *YFP*2 are similar. Moreover, the delay introduced in the third model can be observed comparing waveform *YFP*1 and *YFP*3. However, this delay does prevent the system from reaching expected steady states. Similar results can be observed for the first version of System B (Fig 4B) but not for the second (Fig 4C): while simulations of the behavioural and the ideal response (*YFP*1 and *YFP*2 waveforms) match, the delayed model highlights malfunctions (*YFP*3 waveform). An error arises during the concurrent switch of both internal variables. Such issues are known as critical races and are detailed in Section “*The critical race issue*” of Material and Methods. A first critical race might have occurred after 20 time units (TUs) but the response of the system is not affected. At 40 TUs, the critical race prevents the falling of X1 which jeopardise the complete response of the system. The system never returns to normal behaviour after this failure. Critical races are therefore not temporary effects but lead to long-term malfunctions. A way to reduce the risk of critical race is to encode states with internal variables in a way that only one internal variable changes on each transition. This is the case, for instance, for the first version of system B. However, it is not always possible. The introduction of additional internal variables is sometimes required to meet this condition, but this is likely to increase the constraints on the design, and thus, on the synthesised GRNs, especially when the number of state increases.

### Impact of the inhomogeneity of regulation parameters

**Fig 5:**
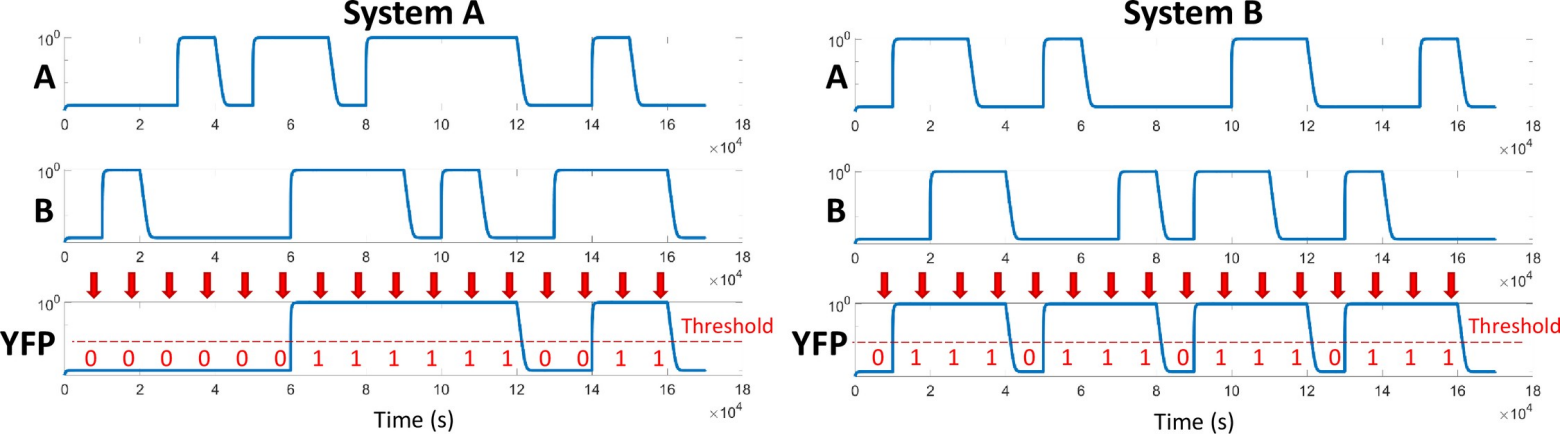
MATLAB simulation results of dynamic models of both systems. (A) corresponds to system A while (B) corresponds to the first version of the system B. The simulation patterns for the inputs are the same as for the Boolean simulation (Fig 4). Thus, for the system A, the response of the dynamic model can be compared to the waveform *YFP*3 in Fig 4A. So does the response of system B and the waveform *YFP*3 in Fig 4B. Concentrations are represented on a logarithmic scale. Red arrows mark the time steps at which the simulated response is sampled, binarised and compared with the expected Boolean response. The dashed line corresponds to the threshold used for binarisation (0.03). The binary values are also given in red below each arrow.

The dynamic models of both systems are simulated with the same stimuli as the Boolean model in Fig 4. One TU in the event-driven simulation corresponds to 1000 seconds to give the system enough time to reach its steady state between each input event. For the system B, only the first version has been considered because the reliability of the second version has already been defeated by the event-driven simulation. Results are given in Fig 5 for both systems. Those are obtained in an ideal case, *i*.*e*. with all parameters at their default value.

**Fig 6:**
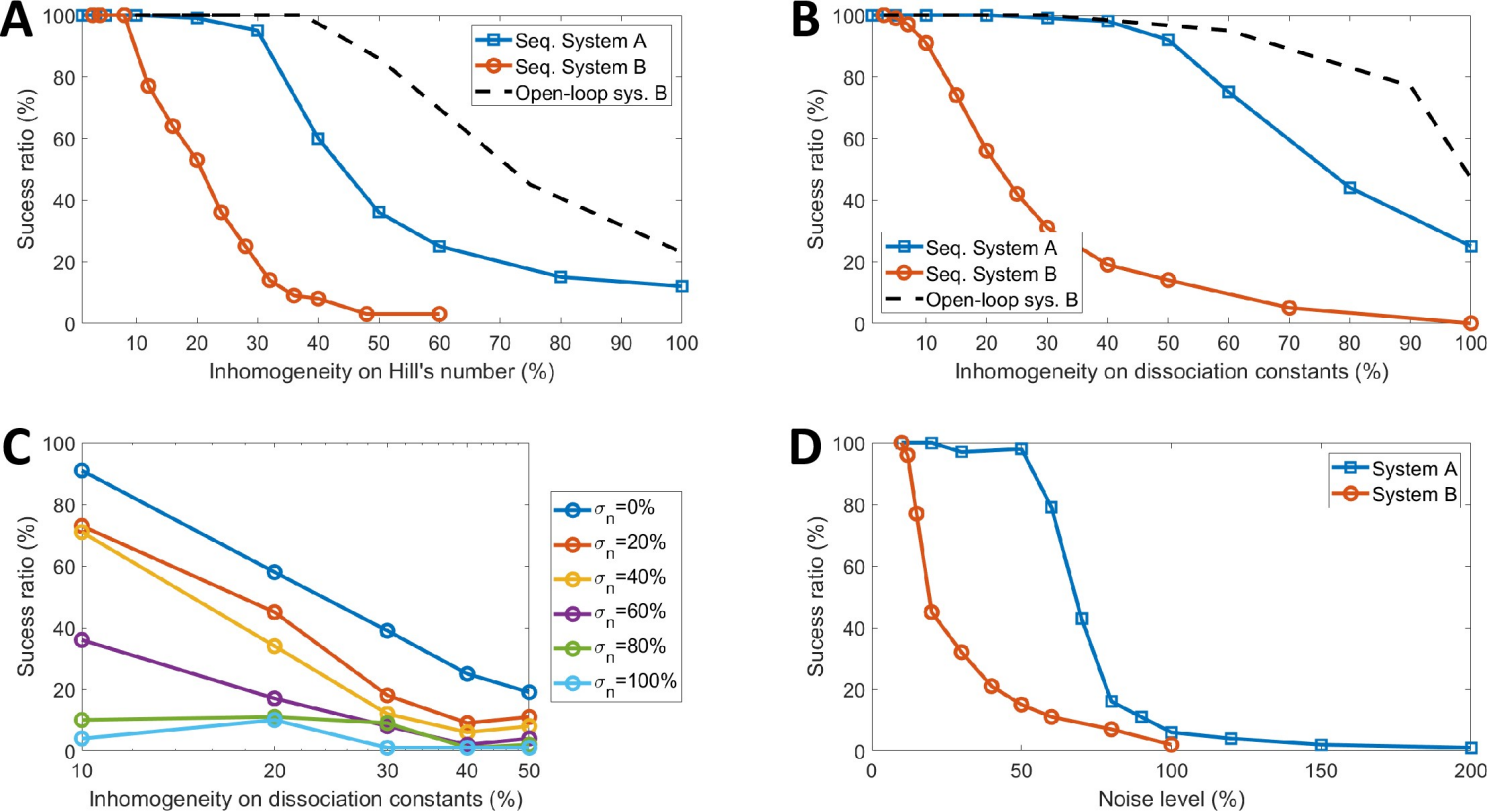
Impact of the parameters inhomogeneity and the stochasticity on the performances of both systems. (A) and (B) are respectively the success ratio as a function of the inhomogeneity on Hill’s number only and on the dissociation constant only for both systems. The success ratio of the open-loop GRN of the System B is also given (dashed black lines). (C) is the success ratio for system B with inhomogeneous Hill’s number and on dissociation constant. Each curve is obtained with a fixed value for the inhomogeneity of Hill’s number and shows the percentage of successful simulations as a function of the inhomogeneity on the dissociation constant. The correspondence between curve colour and the inhomogeneity of Hill’s number is given in the box next to the figure. (D) corresponds to the success ratio as a function of the noise level applied to each transcription and translation mechanisms for both systems.

Then, the methodology described in Section “*Impact of the Parameters Inhomogeneity”* of Material and Methods is applied to assess the impact of the inhomogeneity of the regulation parameter in the performances of the system. The success rate is given as a function of the inhomogeneity value *σ* in Fig 6A for the Hill’s number and Fig 6B for the dissociation constant. First, it can be observed that both systems work properly for a low inhomogeneity but their reliability decreases drastically when the inhomogeneity increases. Moreover, the system with single feedback is less sensitive to inhomogeneity than the system with a double feedback: the system A tolerates up to 20% of inhomogeneity while failing simulations start to occur for the system B at 10% of inhomogeneity.

According to Fig 6C, with 20% of dispersion on both Hill’s number and dissociation constant, the probability of success for the most favourable case (system A) is less than 50%. This observation can be related with experimental results published by Andrews *et al*. [15]. They characterised 10 different NOT gates in *E*.*Coli*, made of 10 different promoters and transcription factors. According to the graphs presented in their paper, we can estimate that the standard deviation of Hill’s dissociation constant in the logarithmic scale is about 30%. According to our simulation, with such inhomogeneity, malfunctions might occur, even for a system with a single internal variable. In [15], 45 toggle switches made by combinations of two among the 10 characterised NOT gates have been experimentally tested and only half of them worked properly.

Two hypotheses can be put forward to explain malfunctions: computation errors in the GRN or synchronisation issues in the feedback loop during state transition. To discriminate between them, the GRN is simulated in open loop (see “*Simulation of the Open-Loop GRN*” section of Material and Methods). Results are plotted only for the more sensitive system (system B) in Fig 6. Malfunction also occurs in the open-loop GRN, but only above 40% of inhomogeneity. Thus, malfunctions above 40% can be explained by the first hypothesis but the ones that occur below 40% are due to synchronisation issues in the feedback loops.

For a deeper understanding of synchronisation issues that may lead to errors during transitions, a failing simulation is dissected in the (S4 File).

### Impact of stochasticity of biological processes

The last step concerns the impact of stochasticity on the stability of the GRNs. The simulation protocol is described in the “*Impact of stochasticity of biological processes*” Section of Material and Methods. The success rate, *i*.*e*. the relative number of successful simulations despite the noise, is given as a function of the noise level in Fig 6D for both systems. Biological noise drastically increases the risk of malfunction. Again, the system with a double feedback is more sensitive than the system with a single one: The corner noise level (noise level above which more than half of the simulations fail) is about 20% for the system B but up to 70% for the system. Additionally, it should be reminded that the theoretical value of *σ* is 100% when all biological mechanisms are Poissonian processes. Consequently, this study predicts that both systems have almost no chance of working because of the biological noise.

The impact of noise on the open-loop GRN is very weak because noise affects the response of GRNs during the transitions but does not prevent them from reaching a steady state. The comparison between complete GRN and open-loop GRN does not make sense regarding the impact of the noise. That is why it is not represented in Fig 6D.

## Discussion

While it is customary to say in electronics that sequential circuits must be designed with great care, this study shows that it is even more true for biology. Simulations of sequential GRNs in an ideal case (*i*.*e*. when regulation parameters are homogeneous) succeed but malfunctions occur when inhomogeneity of regulation parameters and/or biological noise is taken into account. These issues are specific to biological processes. In electronics, elementary parts of circuits are composed of transistors with equivalent static and dynamic performances, which makes the digital electronic circuits robust to noise.

### Reliability

To reduce the risk of malfunctions, the strategy deployed for years in microelectronics is to synchronise transitions in feedback loops. For that purpose, a kind of bandleader signal, namely the clock, is used to regulate the timings during which the next state is computed and during which the feedback is applied. It should be reminded that, in this context, synchronous means that all the clock-sensitive devices have to receive clock ticks at the same time, whatever the delay between two clock ticks. The keystone of synchronous circuits are clock-sensitive memories also called flip-flops or registers [11]. The achievement of such registers in biology has not been widely addressed in the literature, except *in silico*. In 2012, Hoteit *et al*. [42] suggested a biological construct performing flip-flop by a direct transposition of the architecture of an electronic register into a GRN [43]. The resulting system is quite large (7 promoters and involves 10 regulating proteins) and implements light-induced promoters to ensure a clean clock signal. Recently, another approach had been proposed by Lin *et al*. [23]. The complexity is comparable to Hoteit’s construct except that the clock signal is performed by post-transcriptional regulation. Although these works look promising, moving from a single register to a complete synchronous multiple-state automaton is still a long path to go: integrating the required number of artificial genes in a single cell seems unrealistic, even considering the upcoming evolution of synthetic biology technologies. Moreover, each register has to be constructed with an orthogonal set of promoters and transcription factors to avoid crosstalk.

Although *a priori* more robust than biological systems, electronic systems can also experience malfunctions. These faults can be either systematic (*e*.*g*. the critical path issue, hardware breakdown) or random (*e*.*g*. noise). Redundancy and voting mechanisms are commonplace solutions to improve the system’s reliability [44, 45]. The strategy consists of using several circuits with different architectures but performing the same function and comparing their responses. The simplest voting system, i.e. the triple modular redundancy, is composed of three equivalent circuits and a Boolean function that selects the output computed in the majority. The price to pay is a drastic increase in the complexity of the system, which is a major obstacle to the adaptation of such a strategy to GRNs. This is why such an approach is mostly used for critical applications, such as aeronautics and the space industry [46]. Moreover, dedicated computer-aided design tools are now routinely used to optimise the integration of redundancy in electronic systems [47]. The adaptation of such systems to the biological context deserves to be investigated. It is also quite fun to notice that redundancy is implemented natively in species genome, making the phenotypes of these species more robust to genotype mutations [48, 49]. For instance, Wang K. et al. demonstrated the functional redundancy of two transcription factors, Myf5 and myogenin, that play a role in rib cage formation of mice during embryogenesis [50]. Both are activated in a different way during the myogenesis but plays an equivalent role: Myf5-deficient mice die perinatally while mice for which a myogenin complementary DNA has been inserted into the Myf5 locus exhibit a normal development. This feature of biology has already served as an inspiration for the design of fault-tolerant electronic circuits [51].

### Optimisation

One way of optimising sequential GRN would be to deeply analyse the impact of the default regulation parameters on the stability of circuits. In the present study, only one set of default parameters has been tested. This set has been chosen to have, for each regulation, a symmetric and stiff dose-response curve, which could be considered as the most favourable condition to insure the good operation of the complete system but the research of another set of default parameters that make the GRN more reliable would be a nice complementary analysis. Such analysis can be performed by running an optimisation algorithm similar to the one used in [52]. However, two main challenges have to be faced: finding an appropriate relevant and quantitative metric to evaluate each set of default parameters, on the one hand, and computation time, on the other hand. Up to now, the metric is just the number of successful runs, which is an integer and is sensitive to the randomness of the evaluation process (two sets of runs with the same sets of parameters might return different scores). Additionally, the full process for one single set of default parameters takes about one hour on a standard computer. Thus, the computation time for the full process nested in the optimisation loop would take days.

### Feasibility

Besides the reliability issue, the question of the complexity of the synthesised GRNs also arises. A common trend for the implementation of large GRNs is to split the circuit into sub-circuits, to integrate each sub-circuit in different cells and to connect cells using natural or artificial cell-to-cell communication mechanisms, such as quorum sensing [53]. This technique reduces the quantity of artificial gene that has to be inserted in a single cell. However, with cell consortia, the issue of signal synchronisation is even more critical. Recent papers provide some interesting insight on this topic. For instance, coupled oscillators in a multi-cellular network have already been demonstrated [54]. On the other hand, optogenetics might be used to efficiently manage the synchronisation signal (clock in the case of synchronous systems) with light-induced promoters [55].

## Conclusion

To conclude this study, we can say that the implementation of sequential circuits with GRNs is tricky. The paper focuses on one of the existing design methodologies for the sequential-behaviour systems, *i*.*e*. the Huffman-Mealy’s method inspired by electronics. Its adaptation to the context of GRNs has been demonstrated and is valid, at least from a theoretical point of view. However, the study of the system’s reliability shows that the practical implementation of these GRNs might be challenging. The risk of malfunction is considerable and seems to increase with the number of feedback loops to implement. However, a larger number of case studies would be needed to confirm this hypothesis. The heterogeneity of all the regulatory mechanisms involved in the GRN and the intrinsic noise of biological mechanisms are the two main malfunction causes. Strategies can be used to improve the reliability of the system but the price to pay is always an increase in the complexity of the system, which is also an issue to overcome. Even for small automata, this amount of regulation mechanism flirts with the limit of artificial genes that can be integrated into a single cell. For that purpose, splitting and distributing the GRN over several cells need to be considered [10, 56]. Such systems have already proven their efficiency in performing combinatorial functions (i.e. functions for which outputs are unique for a given input combination). The extension of this technique to sequential systems deserves to be explored, both from a theoretical and from an experimental point of view.

## Supporting information

S1 File Detailed description of the design of the system A. This file contains the specification of the system A, the steps of its synthesis with Huffman-Mealy’s method, the construction of the GRN, a possible practical implementation made of elementary part characterised by Shin *et al*. in [32], the three Boolean Models in VHDL, the MATLAB script of the dynamic model and the description of the open-loop GRN.
(PDF)

S2 File Detailed description of the design of the system B. This file contains the specification of the system B, the steps of its synthesis with Huffman-Mealy’s method, the construction of the GRN, a possible practical implementation made of elementary part characterised by Shin *et al*. in [32], the three Boolean Models in VHDL, the MATLAB script of the dynamic model and the description of the open-loop GRN.
(PDF)

S3 File Examples of distribution of parameters used for the assessment of the impact of the parameters inhomogeneity. This file contains examples of parameters distribution and a table with the range of parameters as a function of the inhomogeneity value for Hill’s number and dissociation constant.
(PDF)

S4 File Examples of failing simulations. This file details an example of failing simulation for the system A.
(PDF)

S5 File Sketch for a possible implementation of the GRN for the system A composed of promoters and transcription factors described in [32]. (PDF)

S6 File Sketch for a possible implementation of the GRN for the system B composed of promoters and transcription factors described in [32]. (PDF)
