# Supplementary material for: Feasibility and reliability of sequential logic with gene regulatory networks
Source: PLoS One. 2021 Mar 30;16(3):e0249234. doi: 10.1371/journal.pone.0249234 (PMC8009411; doi:10.1371/journal.pone.0249234)
Supplement: S1 File — This file contains the specification of the system A, the steps of its synthesis with Huffman-Mealy’s method, the construction of the GRN, a possible practical implementation made of elementary part characterised by Shin et al. in [32], the three Boolean Models in VHDL, the MATLAB script of the dynamic model and the description of the open-loop GRN. (PDF) [file pone.0249234.s001.pdf]

Feasibility and reliability of sequential logic with gene regulatory networks

Morgan Madec[1]*, Elise Rosati[1], Christophe Lallement[1]

[1]Laboratory of Engineering Sciences, Computer Sciences and Imaging, UMR 7357 (University of Strasbourg / CNRS), 300, boulevard Sébastien Brandt, 67412 Illkirch, France. *Corresponding author, e-mail: morgan.madec@unistra.fr

---

Supporting Information 1

Design and Simulation of the System A

---

1. Specifications

System A operates as follows: "*The system is composed of two inputs (A and B) and one output (YFP). The output only goes high if both inputs are high. Conversely, the output goes low if both inputs are low. Otherwise, the output stays at its previous state.'*

2. State Diagram

The state diagram of system A is described in Fig. 1. It is composed of six states. Initially, the signals A, B and YFP are low and the system is in state S1. From this initial state, the system can reach S3 if A goes high, or S2 if B goes high. From state S2 (resp. S3), it can then reach back S1 if the active input B (resp. A) returns low, or S4 if the other input A (resp. B) also goes high. Reaching S4 triggers the production of YFP. The lower part of the diagram is the symmetrical of the higher one. When A or B goes down, the system goes to S5 or S6 respectively. From S5 or S6 states, it can go back to S4 if both input get high, or to the initial state S1 if both inputs go low. In this case, YFP also goes low.

3. Synthesis With Huffman-Mealy's Method

The Huffman-Mealy's method is a five-step process that computes the transition logic and the output logic of an asynchronous sequential system described by a state diagram [1]. The state diagram of the system A is given in Fig 1.

3.1. Phase matrix

The first step consists of translating the state diagram into a phase matrix. Each line of this phase matrix corresponds to one state: the stable state is written below the corresponding input combination and

marked up (red-coloured in our case) while the states toward transitions exist are recorded below the input combination that triggers the transition. The phase matrix of system A is given in Table 1.

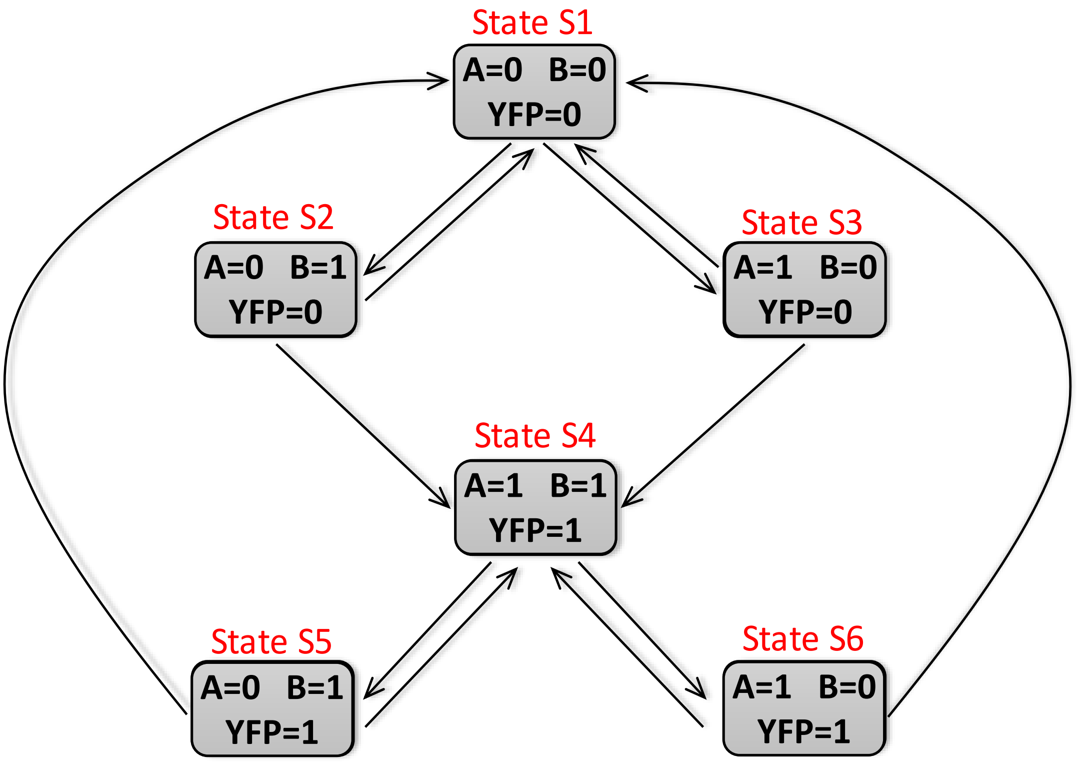

Figure 1. State diagram of system A. The system is composed of six states.

Table 1. Phase matrix for system A.

Stable State	Input combination (AB)				Output YFP
	0 0	0 1	1 1	1 0	
S_1	S_1	S_2		S_3	0
S_2	S_1	S_2	S_4		0
S_3	S_1		S_4	S_3	0
S_4		S_5	S_4	S_6	1
S_5	S_1	S_5	S_4		1
S_6	S_1		S_4	S_6	1

3.2. Reduced phase matrix

The second step consists of reducing the phase matrix by combining lines that are compatible with each other. Two (or more) lines are compatible as soon as they have a common state number for each input combination (if a cell is empty, it is compatible with every state). For the phase matrix of Table 1, it turns out that the first three lines, on the one hand, and the last three lines, on the other hand, are compatible. This process leads to the reduced phase matrix given in Table 2.

Table 2. Reduced phase matrix for system A.

Input combination (AB)			
0 0	0 1	1 1	1 0
S_1	S_2	S_4	S_3
S_1	S_5	S_4	S_6

3.3. State encoding

The third step is the state encoding. First, the number of lines in the reduced phase matrix sets the minimal number of internal variables (and thus the number of positive feedback loop) the system requires. In our case, as there are only two lines in the reduced phase, one internal variable is enough. Let X be this variable. The choice of state encoding is arbitral. Let us choose $X = 0$ for states S1 to S3 and $X = 1$ for states S4 to S6.

3.4. Transition logic Karnaugh map

The fourth step consists of translating the reduced phase matrix into the transition Karnaugh map and solve it. Column headers of the Karnaugh map are the input combinations whereas row headers are the internal variables. It gives the next value of internal variables as a function of the current ones and inputs. The map is filled as following:

- for cells that correspond to stable states (red-coloured states in the reduced phase matrix), the internal variables have to stay at their previous value. Thus, we just have to fill these cells with the same value as in the line header.
- for cells that correspond to transition states (black-coloured states in the reduced phase matrix), internal variables have to change to the combination that makes the state stable. Thus, we just have to fill these cells with the value of the line header for which the state is stable.

The transition Karnaugh map for system A is given in Table 3.

Table 3. Transition Karnaugh map for System A. Values in cells corresponds to the next state of the internal variable.

		Input combination (AB)			
		0 0	0 1	1 1	1 0
Internal Variable (X)	0	0	0	1	0
	1	0	1	1	1

Then, the Boolean equations of the transition logic, which gives the next value of internal variable X' as a function of the current one X and the inputs A and B, are computed from the transition Karnaugh map. In our case, the equation is

$$X' = A \cdot B + X \cdot (A + B) \tag{1}$$

3.5. Output logic Karnaugh map

The last step of Huffman-Mealy's method consists of building and solving the Karnaugh map for the outputs of the system. The output logic Karnaugh map is built in the same way as for the transition logic Karnaugh map (see Section 3.4). However, each cell is filled with the output value corresponding to each state, *i.e.* $YFP = 0$ for states S1 to S3 and $YFP = 1$ for states S4 to S6.

For this example, the output logic Karnaugh map and the transition logic Karnaugh map are similar. But this is not the case in general. The output logic Boolean equation is

$$YFP = A \cdot B + X \cdot (A + B) \tag{2}$$

4. Construction of the GRN

The construction of Gene Regulation Network (GRN) from the Boolean equations is performed with GeNeDA, a genetic design automation tool derived from the field of digital electronics [2]. GeNeDA computes the optimal GRN that matches a Boolean equation by assembling abstracted biological parts (regulated promoters) given in a library. In our case, a library with four abstracted biological parts is used:

- A promoter with a single repressor that performs a NOT function ($\bar{R}$, see Fig. 2A)
- A promoter with one activator and one repressor that performs an INH function ($A \cdot \bar{R}$, see Fig. 2B)
- A promoter with two repressors that performs an NOR function ($\overline{R_1 + R_2}$, see Fig. 2C)
- A promoter with two activators that performs an OR function ($R_1 + R_2$, see Fig. 2D)

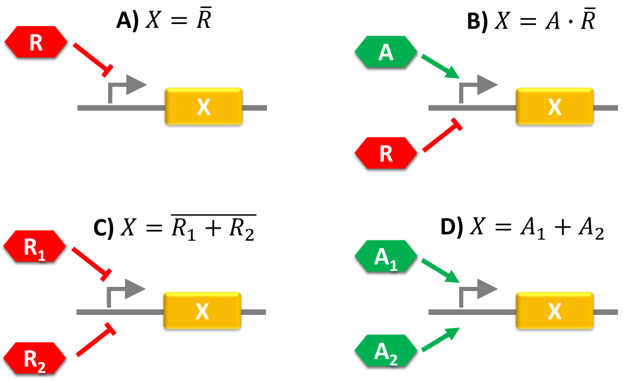

Figure 2. The four abstracted constructs available in the GeNeDA library. A) is a NOT gate, B) is an INH gate, C) is NOT gate, and D) is an OR gate.

The GRN inferred by GeNeDA for equations (1) and (2) is shown in Fig. 3. It is composed of 5 promoters and 5 transcription factors ($R1$ to $R4$ and X which is the internal variable). Equations (3) to (7) are the Boolean equations of each transcription factor and equation (8) is the equation of the output.

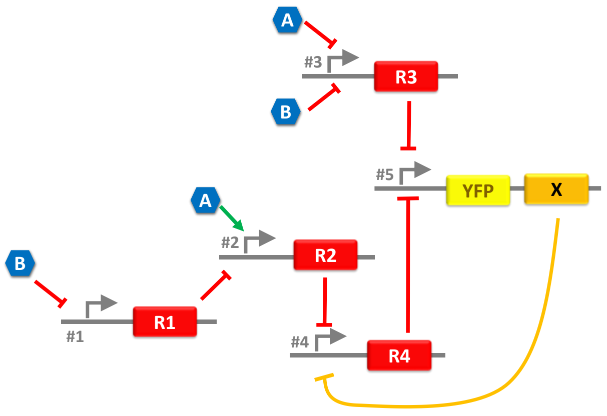

Figure 3. GRN inferred by GeNeDA for Eq. (1) and (2). The GRN is composed of 5 operons and 5 transcription factors.

$$R1 = \bar{B} \tag{3}$$

$$R2 = A \cdot \overline{R1} = A \cdot B \tag{4}$$

$$R3 = \overline{A + B} \tag{5}$$

$$R4 = \overline{R_2 + X} = \overline{(A \cdot B) + X} \tag{6}$$

$$X' = \overline{R_3 + R_4} = \overline{R_3} \cdot \overline{R_4} = (A + B) \cdot [(A \cdot B) + X] = (A \cdot B) + X \cdot (A + B) \quad (7)$$

$$YFP = X' = (A \cdot B) + X \cdot (A + B) \quad (8)$$

5. Possible Concrete Implementation

In 2020, J. Shin *et al*. designed and validated experimentally large GRN in *E.Coli*, composed of eighteen different NOT and NOR constructs, and performing a binary to seven-segment display transcoder [3]. We propose here a possible implementation of the GRN of the system A reinvesting the parts designed by J. Shin *et al*. The correspondence between abstracted transcription factors (R1, R2, etc.) and actual transcription factors (amtR, lmrA, etc.) is the following:

- The input A corresponds to Tet Repressor (*tetR*)
- The input B corresponds to Lux Repressor (*luxR*)
- The transcription factor $R1$ corresponds to the repressor *ameR*
- The transcription factor $R2$ corresponds to the repressor *phlF*
- The transcription factor $R3$ corresponds to the repressor *vanR*
- The transcription factor $R4$ corresponds to the repressor *lmrA*
- The internal variable X corresponds to the repressor *bm3R1*
- As there is no direct INH construct, a new transcription factor *amtR* is required to replace the activation of the promoter #2 by A with a double repression.

The corresponding GRN is shown in Fig 4. A larger sketch is also provided as Supporting Information 5.

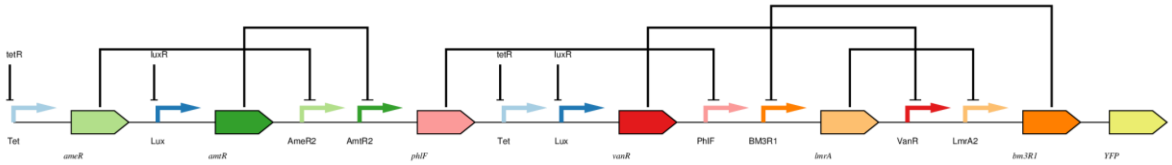

Figure 4. Possible implementation of the GRN associated with the system A using constructs described in [3].

6. Boolean Model

The behaviour of the system at the Boolean level can be described in VHDL, a hardware description language dedicated to the modelling and the simulation of digital electronic circuits [4]. VHDL enables the modelling of a given system at different levels of abstraction. In our case, the system is described at three different levels of abstractions: the behavioural model, the ideal model of the GRN and the delayed model of the GRN. The Listing 1 is the VHDL description of this system.

5.1. Behavioural model

The behavioural model of the system corresponds to a direct and procedural translation in VHDL of the state diagram in VHDL. Thus, it can be considered as a reference model because it describes strictly the targeted behaviour. In the Listing 1, the behavioural model corresponds to the `PROCESS` (that describes the state diagram) and the affectation of `YFP1`.

5.2. Ideal model of the GRN

The ideal model of the GRN is the transcription of equations (7) and (8). In the Listing 1, it corresponds to the affectations of `X2` and `YFP2`. This model can be used to validate the design process.

5.3. Delayed model of the GRN

The delayed model for the GRN is composed of the same equations as the ideal model except that delays are introduced for each regulation process. The description corresponds to the raw form of equations (3) to (7) instead of the compact form of equation (7) in order to introduce the delays at the right place:

- Between a switch of B and the resulting switch of R1,
- Between a switch of R1 or A the resulting possible switch of R2,
- Between a switch of A or B and the resulting possible switch of R3,
- Between a switch of R3 or R4 and the resulting possible switch of X and YFP,
- Between a switch of R2 or X and the resulting possible switch of R4.

Listing 1. VHDL model of system A.

```
LIBRARY IEEE;
USE IEEE.STD_LOGIC_1164.ALL;

ENTITY Ex1 IS
END ENTITY;

ARCHITECTURE Test OF Ex1 IS
   SIGNAL A, B : STD_LOGIC := '0';
   SIGNAL YFP1, YFP2, YFP3 : STD_LOGIC := '0';
   SIGNAL X1, X2, X3 : STD_LOGIC := '0';
   SIGNAL C1, C2, C3, C4,  C5 : STD_LOGIC := '0';
BEGIN

   -- Behavioural model
   PROCESS (A,B)
   BEGIN
      IF (X1='0') THEN
         X1 <= A AND B;
      ELSIF (X1='1') THEN
        X1 <= A OR B;
      END IF;
   END PROCESS;
   YFP1 <= X1;

   -- Ideal model of the GRN
   X2 <= (A AND B) OR (X2 AND (A OR B));
   YFP2 <= X2;

   -- Delayed model of the GRN
   C1 <= NOT(A) AFTER 100 ps;
   C2 <= B AND NOT(C1) AFTER 100 ps;
   C3 <= A OR B AFTER 100 ps;
   C4 <= NOT(X2) AFTER 100 ps;
   C5 <= C3 AND NOT(C4) AFTER 100 ps;
   X3 <= C2 OR C5;
   YFP3 <= X3;

   -- Test Vector
   A <= '0', '1' AFTER 3 ns, '0' AFTER 4 ns, '1' AFTER 5 ns, '0'
        AFTER 7 ns, '1' AFTER 8 ns, '0' AFTER 12 ns, '1' AFTER 14 ns,
        '0' AFTER 15 ns;
   B <= '0', '1' AFTER 1 ns, '0' AFTER 2 ns, '1' AFTER 6 ns, '0'
        AFTER 9 ns, '1' AFTER 10 ns, '0' AFTER 11 ns, '1' AFTER 13
        ns, '0' AFTER 16 ns;
END ARCHITECTURE;
```

5.4. Test vector

The test vector is a description of the stimuli applied to the system in order to validate its correct operation. Ideally, the test vector has to cover all the possible path in the state diagram. For our system, the test vector corresponds to the timing diagram shown in Fig. 5.

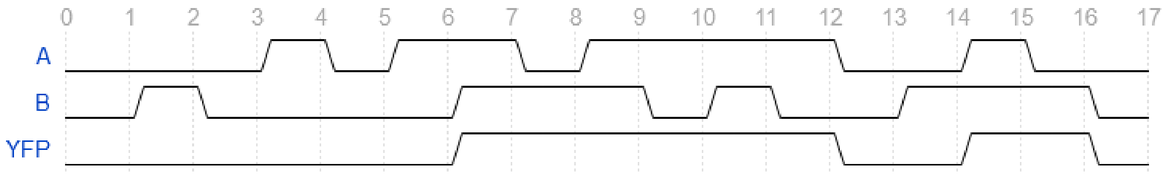

Figure 5. Timing diagram of the test vector and expected response of the system A.

7. Dynamic Model

We established the dynamic models for system A directly from GRN architecture. The dynamic model is composed of a set of 14 ordinary differential equations (ODEs), one for each input (2), one for each transcription factor (5), one for each associated mRNA (5), one for the reporter (1) and one for the mRNA associated with the reporter (1). Regulations are described as a modulation of the transcription rate according to Hill's equation [5].

Equations (9) to (22) are the dynamic model of the system A. Table 4 summarise the parameters of the models. For computation purpose, the concentrations of all involved molecules are gathered in a state vector. Table 5 gives the way the concentrations of molecules are ordered in this state vector. The Listing 2 is the MATLAB function `SystemA_ODE` which computing the derivative terms of the state vector `dY` as a function of the state vector itself `Y` and the time `t`, *i.e.* equations (9) to (22).

The input stimuli are also encoded in `SystemA_ODE` by computing `betaA` and `betaB` as a function of the time.

$$\frac{d[A]}{dt} = \beta_A(t) - d \cdot [A] \tag{9}$$

$$\frac{d[B]}{dt} = \beta_B(t) - d \cdot [B] \tag{10}$$

$$\frac{d[mR1]}{dt} = K_{TR} \cdot \left(\alpha + (1-\alpha) \cdot \frac{K_1^{n_1}}{K_1^{n_1} + [B]^{n_1}} \right) - d_m \cdot [mR1] \tag{11}$$

$$\frac{d[R1]}{dt} = K_{TL} \cdot [mR1] - d \cdot [R1] \tag{12}$$

$$\frac{d[mR2]}{dt} = K_{TR} \cdot \left(\alpha + (1-\alpha) \cdot \frac{K_2^{n_2}}{K_2^{n_2} + [R1]^{n_2}} \cdot \frac{[A]^{n_3}}{K_3^{n_3} + [A]^{n_3}} \right) - d_m \cdot [mR2] \tag{13}$$

$$\frac{d[R2]}{dt} = K_{TL} \cdot [mR2] - d \cdot [R2] \tag{14}$$

$$\frac{d[mR3]}{dt} = K_{TR} \cdot \left(\alpha + (1-\alpha) \cdot \frac{K_4^{n_4}}{K_4^{n_4} + [A]^{n_4}} \cdot \frac{K_5^{n_5}}{K_5^{n_5} + [B]^{n_5}} \right) - d_m \cdot [mR3] \tag{15}$$

$$\frac{d[R3]}{dt} = K_{TL} \cdot [mR3] - d \cdot [R3] \tag{16}$$

$$\frac{d[mR4]}{dt} = K_{TR} \cdot \left(\alpha + (1-\alpha) \cdot \frac{K_6^{n_6}}{K_6^{n_6} + [R2]^{n_6}} \cdot \frac{K_7^{n_7}}{K_7^{n_7} + [X]^{n_7}} \right) - d_m \cdot [mR4] \tag{17}$$

$$\frac{d[R4]}{dt} = K_{TL} \cdot [mR4] - d \cdot [R4] \tag{18}$$

$$\frac{d[mX]}{dt} = K_{TR} \cdot \left(\alpha + (1-\alpha) \cdot \frac{K_8^{n_8}}{K_8^{n_8} + [R3]^{n_8}} \cdot \frac{K_9^{n_9}}{K_9^{n_9} + [R4]^{n_9}} \right) - d_m \cdot [mX] \tag{19}$$

$$\frac{d[X]}{dt} = K_{TL} \cdot [mX] - d \cdot [X] \tag{20}$$

$$\frac{d[mYFP]}{dt} = K_{TR} \cdot \left(\alpha + (1-\alpha) \cdot \frac{K_8^{n_8}}{K_8^{n_8} + [R3]^{n_8}} \cdot \frac{K_9^{n_9}}{K_9^{n_9} + [R4]^{n_9}} \right) - d_m \cdot [mYFP] \tag{21}$$

$$\frac{d[YFP]}{dt} = K_{TL} \cdot [mYFP] - d \cdot [YFP] \tag{22}$$

Listing 2. MATLAB function giving the dynamic model of system A.

```
function dY = SystemA_ODE(t,Y)

  % Nominal value of parameters
  global Ktr alpha K_nom n_nom Ktl dm d

  % Variations
  global sigma_K sigma_n sigma_noise

  % Waveform definition
  global tf
  betaA = 0;
  if (t>3*tf && t<4*tf) || (t>5*tf && t<7*tf) || (t>8*tf && t<12*tf)
     || (t>14*tf && t<15*tf)
    betaA = 1e-3;
  end

  betaB = 0;
  if (t>1*tf && t<2*tf) || (t>6*tf && t<9*tf) || (t>10*tf && t<11*tf)
     || (t>13*tf && t<16*tf)
    betaB = 1e-3;
  end

  % Parameter set
  n = max(ones(1,9)*n_nom .* (1+sigma_n*randn(1,9)),0);
  while sum(n>0)<9,
    n = max(ones(1,9)*n_nom .* (1+sigma_n*randn(1,9)),0);
  end
  K = 10.^(log10(ones(1,9)*K_nom) .* (1+sigma_K*randn(1,9)));

  % Equations
  dY(1)  = betaA - d*Y(1);
  dY(2)  = betaB - d*Y(2);
  dY(3)  = Ktr*Hill_R(K(1),n(1),Y(2)) - dm*Y(3);
  dY(4)  = Ktl*Y(3) - d*Y(4);
  dY(5)  = Ktr*Hill_AR(K(3),n(3),Y(1),K(2),n(2),Y(4)) - dm*Y(5);
  dY(6)  = Ktl*Y(5) - d*Y(6);
  dY(7)  = Ktr*Hill_RR(K(4),n(4),Y(1),K(5),n(5),Y(2)) - dm*Y(7);
  dY(8)  = Ktl*Y(7) - d*Y(8);
```

```
dY(9)  = Ktr*Hill_RR(K(6),n(6),Y(6),K(7),n(7),Y(12)) - dm*Y(9);
dY(10) = Ktl*Y(9) - d*Y(10);
dY(11) = Ktr*Hill_RR(K(8),n(8),Y(8),K(9),n(9),Y(10)) - dm*Y(11);
dY(12) = Ktl*Y(11) - d*Y(12);
dY(13) = Ktr*Hill_RR(K(8),n(8),Y(8),K(9),n(9),Y(10)) - dm*Y(13);
dY(14) = Ktl*Y(13) - d*Y(14);

dY = dY' + randn(14,1)*sigma_noise;
```

Table 4. List of specific parameters.

Symbol	Description
K_1	Dissociation constant of B on the promoter of the operon #1
K_2	Dissociation constant of R1 on the promoter of the operon #2
K_3	Dissociation constant of A on the promoter of the operon #2
K_4	Dissociation constant of A on the promoter of the operon #3
K_5	Dissociation constant of B on the promoter of the operon #3
K_6	Dissociation constant of R2 on the promoter of the operon #4
K_7	Dissociation constant of X on the promoter of the operon #4
K_8	Dissociation constant of R3 on the promoter of the operon #5
K_9	Dissociation constant of R4 on the promoter of the operon #5
n_1	Hill's number for the repression of the promoter of the operon #1 by B
n_2	Hill's number for the repression of the promoter of the operon #2 by R1
n_3	Hill's number for the activation of the promoter of the operon #2 by A
n_4	Hill's number for the repression of the promoter of the operon #3 by A
n_5	Hill's number for the repression of the promoter of the operon #3 by B
n_6	Hill's number for the repression of the promoter of the operon #4 by R2
n_7	Hill's number for the repression of the promoter of the operon #4 by X
n_8	Hill's number for the repression of the promoter of the operon #5 by R3
n_9	Hill's number for the repression of the promoter of the operon #5 by R4

Table 5. Composition of the state vector.

Index	Description	Description
1	$[A]$	Concentration of A
2	$[B]$	Concentration of B
3	$[mR1]$	Concentration of mRNA for R1
4	$[R1]$	Concentration of R1
5	$[mR2]$	Concentration of mRNA for R2
6	$[R2]$	Concentration of R2
7	$[mR3]$	Concentration of mRNA for R3
8	$[R3]$	Concentration of R3
9	$[mR4]$	Concentration of mRNA for R4
10	$[R4]$	Concentration of R4
11	$[mX]$	Concentration of mRNA for X
12	$[X]$	Concentration of X
13	$[mYFP]$	Concentration of mRNA for YFP
14	$[YFP]$	Concentration of YFP

8. Open-loop GRN

The open-loop GRN is obtained by deleting the repression of the operon #4 by X and adding a new input X_o that represses the operon #4 directly. The sketch of this new GRN is shown in Fig. 6. The timing diagram used for the simulation of the open-loop GRN is given in Fig. 7.

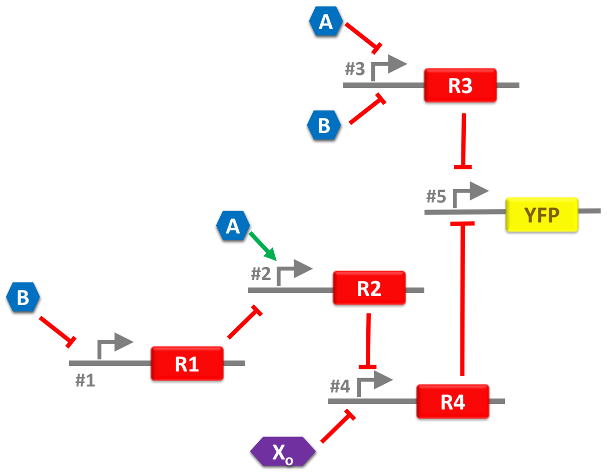

Figure 6. Sketch of the open-loop GRN. This GRN is now composed of three inputs and one output. Its associated Boolean function is equation (2)

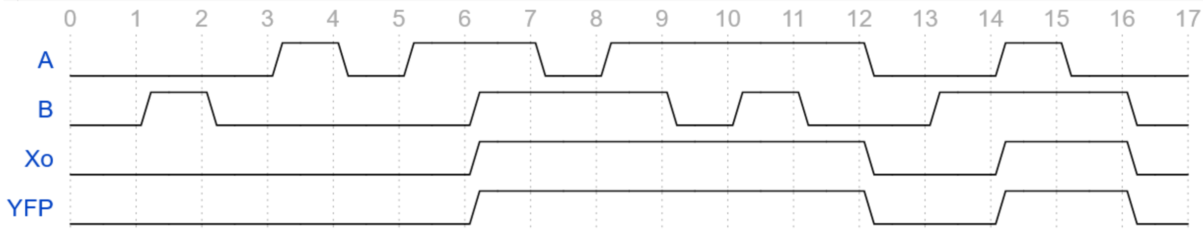

Figure 7. Timing diagram of the test vector and expected response of the system A in an open loop.

9. References

1. Micheli GD. Synthesis and optimization of digital circuits. McGraw-Hill Higher Education; 1994.

2. Madec M, Pecheux F, Gendrault Y, Rosati E, Lallement C, Haiech J. GeNeDA: An Open-Source Workflow for Design Automation of Gene Regulatory Networks Inspired from Microelectronics. Journal of Computational Biology. 2016;23. doi:10.1089/cmb.2015.0229

3. Shin J, Zhang S, Der BS, Nielsen AA, Voigt CA. Programming Escherichia coli to function as a digital display. Molecular Systems Biology. 2020;16: e9401. doi:10.15252/msb.20199401

4. Ashenden PJ. The Designer's Guide to VHDL. Morgan Kaufmann; 2010.

5. Konkoli Z. Safe uses of Hill's model: an exact comparison with the Adair-Klotz model. Theoretical biology & medical modelling. 2011;8: 10–10. doi:10.1186/1742-4682-8-10
