# Supplementary material for: Feasibility and reliability of sequential logic with gene regulatory networks
Source: PLoS One. 2021 Mar 30;16(3):e0249234. doi: 10.1371/journal.pone.0249234 (PMC8009411; doi:10.1371/journal.pone.0249234)
Supplement: S2 File — This file contains the specification of the system B, the steps of its synthesis with Huffman-Mealy’s method, the construction of the GRN, a possible practical implementation made of elementary part characterised by Shin et al. in [32], the three Boolean Models in VHDL, the MATLAB script of the dynamic model and the description of the open-loop GRN. (PDF) [file pone.0249234.s002.pdf]

Feasibility and reliability of sequential logic with gene regulatory networks

Morgan Madec[1]*, Elise Rosati[1], Christophe Lallement[1]

[1]Laboratory of Engineering Sciences, Computer Sciences and Imaging, UMR 7357 (University of Strasbourg / CNRS), 300 boulevard Sébastien Brandt, 67412 Illkirch, France. *Corresponding author, e-mail: morgan.madec@unistra.fr

Supporting Information 2

Design and Simulation of the System B

1. Specifications

System B operates as follows: "*the system is composed of two inputs (A and B) and one output (YFP). The system reacts on any set of two consecutive pulses on A and/or B. The output YFP rises at the beginning of the first pulse and falls at the end of the second pulse. Four possible scenarios have to be considered: i) both pulses are performed with the same input, ii) pulses are performed with different inputs but the second pulse starts after the end of the first one, iii) pulses are performed with different inputs and the second pulse starts before the end of the first one and iv) pulses are performed with different inputs and the second pulse takes place entirely during the first one.*"

2. State Diagram

The state diagram of the system B is described in Fig. 1. It is composed of seven states. Initially, A, B, and YFP are low and the system is in state S1. From this initial state, the system can reach S3 if A goes high, or S2 if B goes high. Then, from state S2 (resp. S3), the system can evolve in two ways depending on the overlapping of the two pulses. If the pulse on A (resp. B) stops before the pulse on B (resp. A) starts, the system goes to state S7 (resp. S6) via S5. Otherwise, if pulses overlap (*i.e.* the second pulse starts before the end of the first), the system also reaches state S7 (resp. S6) but via S4 instead. Ultimately, whether in S6 or S7, the system goes back to S1 at the end of the second pulse.

3. Synthesis With Huffman-Mealy's Method

The Huffman-Mealy's method is a five-step process that computes the transition logic and the output logic of an asynchronous sequential system described by a state diagram [1]. The state diagram of the System B is given in Fig. 1.

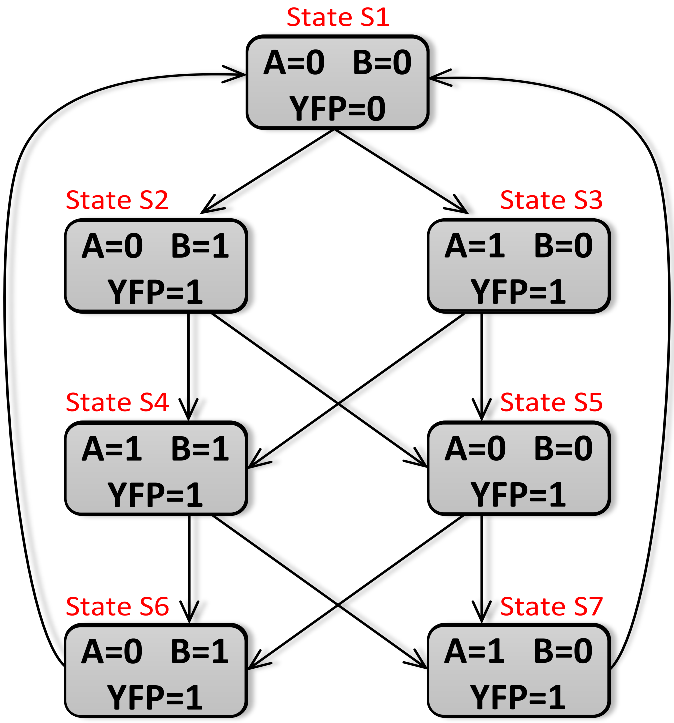

Figure 1. State diagram of System B. The system is composed of seven states. Values given in each state correspond to A and B on the left side of the slash character and YFP on the right side.

3.1. Phase matrix

The first step consists of translating the state diagram into a phase matrix. Each line of this phase matrix corresponds to one state: the stable state is written below the corresponding input combination and marked up (red-coloured in our case) while the states toward transitions exist are recorded below the input combination that triggers the transition. The phase matrix of system B is given in Table 1.

Table 1. Phase matrix for system B.

Stable State	Input combination (AB)				Output
	0 0	0 1	1 1	1 0	YFP
S_1	S_1	S_2		S_3	0
S_2	S_5	S_2	S_4		1
S_3	S_5		S_4	S_3	1
S_4		S_6	S_4	S_7	1
S_5	S_5	S_6		S_7	1
S_6	S_1	S_6			1
S_7	S_1			S_7	1

3.2. Reduced phase matrix

The second step consists of reducing the phase matrix by combining lines that are compatible with each other. Two (or more) lines are compatible as soon as they have a common state number for each

input combination (if a cell is empty, it is compatible with every state). For the phase matrix of Table 1, it turns out that the first cannot be combined with any other line, lines 2 and 3 can be combined together, lines 4 and 5 can be combined together and lines 6 and 7 can be combined together. This process leads to the reduced phase matrix given in Table 2.

Table 2. Reduced phase matrix for system B.

Input combination (AB)			
0 0	0 1	1 1	1 0
S_1	S_2		S_3
S_5	S_2	S_4	S_3
S_5	S_6	S_4	S_7
S_1	S_6		S_7

3.3. State encoding

The third step is the state encoding. First, the number of lines in the reduced phase matrix sets the minimal number of internal variables (and thus the number of positive feedback loop) the system requires. In this case, there are four lines in the reduced phase. Thus, two internal variables are required. Let X and Y be these variables. The choice of state encoding is arbitral. For this system, two alternative states encoding are tested. In the first version, called "*Gray encoding*" in the following, $XY = 00$ encodes for states S1, $XY = 01$ for states S2 and S3, $XY = 11$ for states S4 and S5 and $XY = 10$ for states S6 and S7. In the second version, called "*binary encoding*" in the following, $XY = 00$ encodes for states S1, $XY = 01$ for states S2 and S3, $XY = 10$ for states S4 and S5 and $XY = 11$ for states S6 and S7.

3.4. Transition logic Karnaugh map

The fourth step consists of translating the reduced phase matrix into the transition Karnaugh map and solve it. Column headers of the Karnaugh map are the input combinations whereas row headers are the internal variables. It gives the next value of internal variables as a function of the current ones and inputs. The map is filled as following:

- for cells that correspond to stable states (red-coloured states in the reduced phase matrix), the internal variables have to stay at their previous value. Thus, we just have to fill these cells with the same value as in the line header.
- for cells that correspond to transition states (black-coloured states in the reduced phase matrix), internal variables have to change to the combination that makes the state stable. Thus, we just have to fill these cells with the value of the line header for which the state is stable.

The transition Karnaugh map for the two versions of the system B is given in Table 3 (Gray encoding) and Table 4 (binary encoding).

Table 3. Transition Karnaugh map for System B with Gray encoding. Values in cells corresponds to the next state of the internal variable. The “don’t care”, *i.e.* “-“, in the cell means that the next value can be either 0 or 1.

		Input combination (AB)			
		0 0	0 1	1 1	1 0
Internal Variables (XY)	0 0	0 0	0 1	- -	0 1
	0 1	1 1	0 1	1 1	0 1
	1 1	1 1	1 0	1 1	1 0
	1 0	0 0	1 0	- -	1 0

Table 4. Transition Karnaugh map for the System B with binary encoding. Values in cells corresponds to the next state of the internal variable. The “don’t care”, *i.e.* “-“, in the cell means that the next value can be either 0 or 1.

		Input combination (AB)			
		0 0	0 1	1 1	1 0
Internal Variables (XY)	0 0	0 0	0 1	- -	0 1
	0 1	1 0	0 1	1 0	0 1
	1 1	0 0	1 1	- -	1 1
	1 0	1 0	1 1	1 0	1 1

Then, the Boolean equations of the transition logic, which gives the next value of internal variables (X’ and Y’) as a function of the current ones (X and Y) and the inputs (A and B), are computed from the transition Karnaugh map. For the Gray encoding version, the equations are

$$X' = Y \cdot (\bar{A} \cdot \bar{B} + A \cdot B) + X \cdot (\bar{A} \cdot B + A \cdot \bar{B}) \tag{1}$$

$$Y' = Y \cdot (\bar{A} \cdot \bar{B} + A \cdot B) + \bar{X} \cdot (\bar{A} \cdot B + A \cdot \bar{B}) \tag{2}$$

For the Gray binary version, the equations are

$$X' = \bar{A} \cdot \bar{B} \cdot \bar{X} \cdot Y + X \cdot (A + B) + A \cdot B + X \cdot \bar{Y} \tag{3}$$

$$Y' = \bar{A} \cdot \bar{B} + A \cdot B \tag{4}$$

3.5. Output logic Karnaugh map

The last step of Huffman-Mealy’s method consists of building and solving the Karnaugh map for the outputs of the system. The output logic Karnaugh map is built in the same way as for the transition logic Karnaugh map (see Section 3.4). However, each cell is filled with the output value corresponding to each state, *i.e.* $YFP = 0$ for state S1 and $YFP = 1$ for states S2 to S7.

The output logic Karnaugh map leads to the following equation for the output logic (valid for both state encodings):

$$YFP = X + Y \quad (5)$$

4. Construction of the GRN

The construction of Gene Regulation Network (GRN) from the Boolean equations is performed with GeNeDA, a genetic design automation tool derived from the field of digital electronics [2]. GeNeDA computes the optimal GRN that matches a Boolean equation by assembling abstracted biological parts (regulated promoters) given in a library. In our case, a library with four abstracted biological parts is used:

- A promoter with a single repressor that performs a NOT function ($\bar{R}$, see Fig. 2A)
- A promoter with one activator and one repressor that performs an INH function ($A \cdot \bar{R}$, see Fig. 2B)
- A promoter with two repressors that performs an NOR function ($\overline{R_1 + R_2}$, see Fig. 2C)
- A promoter with two activators that performs an OR function ($R_1 + R_2$, see Fig. 2D)

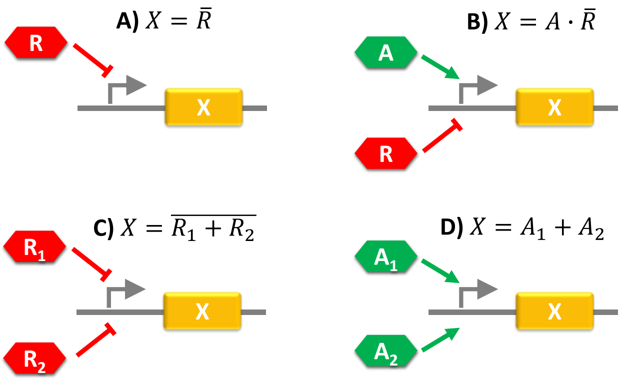

Figure 2. The four abstracted constructs available in the GeNeDA library. A) is a NOT gate, B) is an INH gate, C) is NOT gate, and D) is an OR gate.

4.1. GRN for the Gray encoding version

The GRN inferred by GeNeDA for equations (1), (2) and (5) is shown in Fig. 3. It is composed of eight promoters and five transcription factors ($R1$, $R2$, $A3$ and the internal variables X and Y). Equations (6) to (10) are the Boolean equations of each transcription factor and equation (11) is the equation of the output.

$$R1 = A \cdot \bar{B} + \bar{A} \cdot B \quad (6)$$

$$R2 = \overline{R1} = A \cdot B + \bar{A} \cdot \bar{B} \quad (7)$$

$$X' = X \cdot \overline{R2} + Y \cdot \overline{R1} = X \cdot (A \cdot \bar{B} + \bar{A} \cdot B) + Y \cdot (A \cdot B + \bar{A} \cdot \bar{B}) \quad (8)$$

$$A3 = \bar{X} \quad (9)$$

$$Y' = A3 \cdot \overline{R2} + Y \cdot \overline{R1} = \bar{X} \cdot (A \cdot \bar{B} + \bar{A} \cdot B) + Y \cdot (A \cdot B + \bar{A} \cdot \bar{B}) \quad (10)$$

$$YFP = X + Y \quad (11)$$

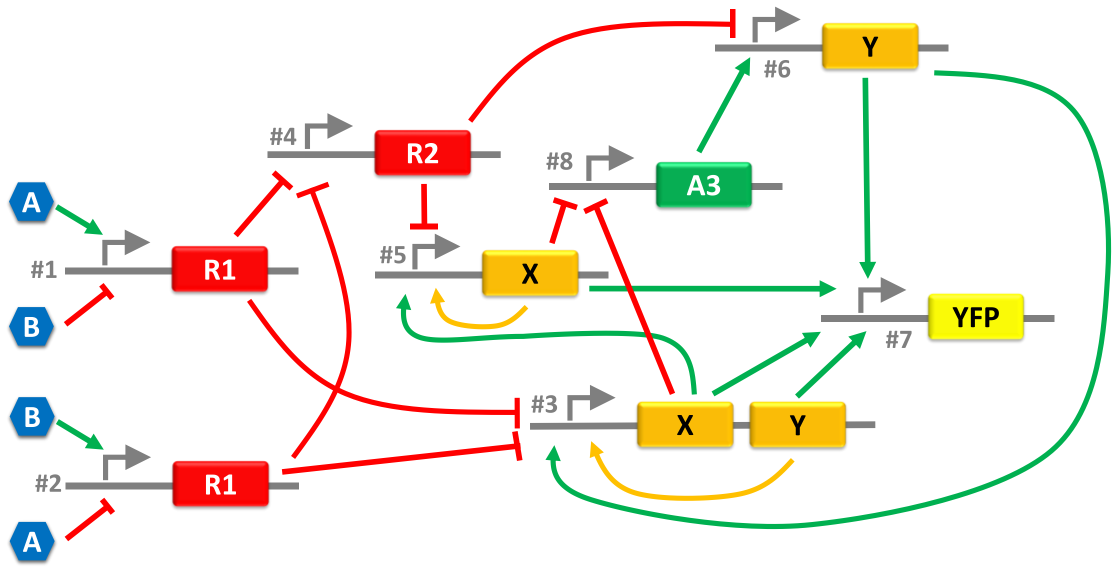

Figure 3. GRN inferred by GeNeDA for Eq. (1), (2), and (5). The GRN is composed of 8 operons and 5 transcription factors.

4.2. GRN for the binary encoding version

The GRN inferred by GeNeDA for equations (3) to (5) is composed of 8 promoters and 6 transcription factors ($R1$, $R2$, $R3$ and the internal variables X and Y). Equations (12) to (16) are the Boolean equations of each transcription factor and equation (17) is the equation of the output. The sketch of the GRN is not shown.

$$Y' = A \cdot \bar{B} + \bar{A} \cdot B \tag{12}$$

$$R1 = \overline{Y'} \tag{13}$$

$$R2 = \bar{A} \tag{14}$$

$$R3 = A + B + X \tag{15}$$

$$\begin{aligned} X' &= X \cdot \overline{R1} + B \cdot \overline{R2} + Y \cdot \overline{R3} + X \cdot \bar{Y} \\ &= X \cdot (A \cdot \bar{B} + \bar{A} \cdot B) + \; B \cdot A + Y \cdot \overline{A + B + X} + X \cdot \bar{Y} \end{aligned} \tag{16}$$

$$YFP = X + Y \tag{17}$$

5. Possible Concrete Implementation

In 2020, J. Shin *et al*. designed and validated experimentally large GRN in *E.Coli*, composed of eighteen different NOT and NOR constructs, and performing a binary to seven-segment display transcoder [3]. We propose here a possible implementation of the GRN of the system A reinvesting the parts designed by J. Shin *et al*. For the Gray encoding version, the correspondence between abstracted transcription factors (R1, R2, etc.) and actual transcription factors (amtR, lmrA, etc.) is the following:

- The input A corresponds to Tet Repressor (*tetR*)
- The input B corresponds to Lux Repressor (*luxR*)
- The transcription factor $R1$ corresponds to the repressor *hlylIR*
- The transcription factor $R2$ corresponds to the repressor *lmrA*
- The internal variable X corresponds to the repressor *vanR*
- The internal variable Y corresponds to the repressor *phlF*

- As there is no direct INH construct, a new transcription factor *betI* is required to replace the activation of the promoter #1 by A with a double repression.
- For the same reason, a new transcription factor *cymR* is required to replace the activation of the promoter #2 by B with a double repression.
- For the same reason, a new transcription factor *ameR* is required to replace the activation of the promoters #5 and #7 by X with a double repression.
- For the same reason, a new transcription factor *atmR* is required to replace the activation of the promoters #3 and #7 by Y with a double repression.
- Ultimately, the transcription factor A3 is not implemented but it is replaced by a direct repression of the promoter #6 by X.

The corresponding GRN is shown in Fig 4. A larger sketch is also provided as Supporting Information 6.

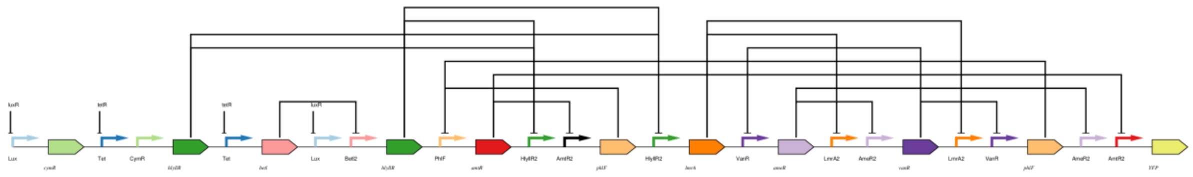

Figure 4. Possible implementation of the GRN associated with the system B using constructs described in [3].

For the correspondence for the binary encoding version is not shown.

6. Boolean Model

The behaviour of the system at the Boolean level can be described in VHDL, a hardware description language dedicated to the modelling and the simulation of digital electronic circuits [4]. VHDL enables the modelling of a given system at different levels of abstraction. In our case, the system is described at three different levels of abstractions: the behavioural model, the ideal model of the GRN and the delayed model of the GRN. The Listing 1 is the VHDL description of this system with the Gray encoding method whereas the Listing 2 corresponds to the binary encoding method.

5.1. Behavioural model

The behavioural model of the system corresponds to a direct and procedural translation in VHDL of the state diagram in VHDL. Thus, it can be considered as a reference model because it describes strictly the targeted behaviour. In both listings, the behavioural model corresponds to the **`PROCESS`** (that describes the state diagram) and the affectation of `YFP1`.

5.2. Ideal model of the GRN

The ideal model of the GRN is the transcription of equations (8), (10) and (11) for the Listing 1 and (12), (16) and (17) for the Listing 2. In both listings, it corresponds to the affectations of `X2` , `Y2` and `YFP2`. This model can be used to validate the design process.

5.3. Delayed model of the GRN

The delayed model for the GRN is composed of the same equations as the ideal model except that delays are introduced for each regulation process. For the Listing 1, the description corresponds to the raw form of equations (6) to (11) in order to introduce the delays at the right place:

- Between a switch of A or B and the resulting possible switch of R1,
- Between a switch of R1 and the resulting switch of R2,
- Between a switch of R2 or X and the possible update of X,

- Between a switch of R1 or Y and the possible update of Y,
- Between a switch of X and the resulting switch of A3,
- Between a switch of R2 or A3 and the resulting possible switch of Y,
- Between a switch of X or Y and the resulting possible switch of YFP.

Listing 1. VHDL model of Gray encoding version of the System B

```
LIBRARY IEEE;
USE IEEE.STD_LOGIC_1164.ALL;

ENTITY Ex2a IS
END ENTITY;

ARCHITECTURE Test OF Ex2a IS

   SIGNAL A, B : STD_LOGIC := '0';
   SIGNAL X1, Y1, X2, Y2, X3, Y3 : STD_LOGIC := '0';
   SIGNAL YFP1, YFP2, YFP3 : STD_LOGIC := '0';
   SIGNAL C1, C2, C3, C4,  C5, C6, C7, C8, C9, C10 : STD_LOGIC;

BEGIN

   -- Behavioural model
   PROCESS (A,B,X1,Y1)
   BEGIN
      IF (X1='0' AND Y1='0') THEN
        IF  ((A OR B) = '1') THEN
           X1 <= '0';
           Y1 <= '1';
        END IF;
      ELSIF (X1='0' AND Y1='1') THEN
        IF ((A XOR B) = '0') THEN
           X1 <= '1';
           Y1 <= '1';
        END IF;
      ELSIF (X1='1' AND Y1='1') THEN
        IF ((A XOR B)='1') THEN
           X1 <= '1';
           Y1 <= '0';
        END IF;
      ELSE
        IF ((A OR B)='0') THEN
          X1 <= '0';
          Y1 <= '0';
        END IF;
    END IF;
   END PROCESS;
   YFP1 <= X1 OR Y1;

   -- Ideal model of the GRN
   X2 <= (Y2 AND NOT(A XOR B)) OR (X2 AND (A XOR B));
   Y2 <= (Y2 AND NOT(A XOR B)) OR (NOT(X2) AND (A XOR B));
   F2 <= X2 OR Y2;

   -- Delayed model of the GRN
   C1 <= A AND NOT(B) AFTER 200 ps;
   C2 <= B AND NOT(A) AFTER 200 ps;
   C3 <= C1 OR C2;
   C4 <= NOT(C3) AFTER 200 ps;
```

```
    C5 <= (Y3 AND NOT(C3)) AFTER 200 ps;
    C6 <= (X3 AND NOT(C4)) AFTER 200 ps;
    C7 <= NOT(X3) AFTER 200 ps;
    C8 <= (C7 AND NOT(C4)) AFTER 200 ps;
    X3 <= C5 OR C6;
    Y3 <= C5 OR C8;
    F3 <= X3 OR Y3 AFTER 200 ps;

    -- Test vector
    A <= '0', '1' AFTER 1 ns, '0' AFTER 3 ns, '1' AFTER 5 ns, '0'
         AFTER 6 ns, '1' AFTER 10 ns, '0' AFTER 12 ns, '1' AFTER 15
         ns, '0' AFTER 16 ns;
    B <= '0', '1' AFTER 2 ns, '0' AFTER 4 ns, '1' AFTER 7 ns, '0'
         AFTER 8 ns, '1' AFTER 9 ns, '0' AFTER 11 ns, '1' AFTER 13 ns,
         '0' AFTER 14 ns;

END ARCHITECTURE;
```

For the Listing 2, the description corresponds to the raw form of equations (12) to (17) in order to introduce the delays at the right place:

- Between a switch of A or B and the resulting possible switch of Y,
- Between a switch of Y and the resulting switch of R1,
- Between a switch of A and the resulting switch of R3,
- Between a switch of B and the resulting switch of R3,
- Between a switch of X and the resulting switch of R3,
- Between a switch of R1 or X and the possible update of X,
- Between a switch of B or R2 and the resulting possible switch of X,
- Between a switch of Y or R3 and the resulting possible switch of X,
- Between the switch of X or Y and the possible update of X.
- Between the switch of X or Y and the possible switch of YFP.

Listing 2. VHDL model of binary encoding version of the System B

```
LIBRARY IEEE;
USE IEEE.STD_LOGIC_1164.ALL;

ENTITY Ex2b IS
END ENTITY;

ARCHITECTURE Test OF Ex2b IS
    SIGNAL A, B : STD_LOGIC := '0';
    SIGNAL X1, Y1, X2, Y2, X3, Y3 : STD_LOGIC := '0';
    SIGNAL F1, F2, F3 : STD_LOGIC := '0';
    SIGNAL C1, C2, C3, C4,  C5, C6, C7, C8, C9, C10 : STD_LOGIC;
BEGIN

    -- Behavioural model
    PROCESS (A,B,X1,Y1)
    BEGIN
       IF (X1='0' AND Y1='0') THEN
         IF  ((A OR B) = '1') THEN
            X1 <= '0';
            Y1 <= '1';
         END IF;
       ELSIF (X1='0' AND Y1='1') THEN
         IF ((A XOR B) = '0') THEN
```

```
          X1 <= '1';
          Y1 <= '1';
       END IF;
     ELSIF (X1='1' AND Y1='1') THEN
       IF ((A XOR B)='1') THEN
          X1 <= '1';
          Y1 <= '0';
       END IF;
     ELSE
       IF ((A OR B)='0') THEN
         X1 <= '0';
         Y1 <= '0';
       END IF;
   END IF;
  END PROCESS;
  F1 <= X1 OR Y1;

  -- Ideal model of the GRN
  X2 <= (NOT(A) AND NOT(B) AND NOT(X2) AND Y2) OR (X2 AND NOT(Y2))
        OR (X2 AND (A XOR B)) OR (A AND B);
  Y2 <= A XOR B;
  F2 <= X2 OR Y2;

  -- Delayed model of the GRN
  C1  <= (A AND NOT(B)) AFTER 200 ps;
  C2  <= (B AND NOT(A)) AFTER 200 ps;
  C3  <= C1 OR C2;
  C4  <= NOT(C3) AFTER 200 ps;
  C5  <= X3 AND NOT(C4) AFTER 200 ps;
  C6  <= NOT(A) AFTER 200 ps;
  C7  <= (B AND NOT(C6)) AFTER 200 ps;
  C8  <= A AFTER 200 ps;
  C9  <= B AFTER 200 ps;
  C10 <= C8 OR C9 OR X3;
  C11 <= (Y3 AND NOT(C10)) AFTER 200 ps;
  C12 <= (X3 AND NOT(Y3)) AFTER 200 ps;
  X3 <= C5 OR C7 OR C12 OR C11;
  Y3 <= C3;
  F3 <= X3 OR Y3 AFTER 200 ps;

  -- Test vector
  A <= '0', '1' AFTER 1 ns, '0' AFTER 3 ns, '1' AFTER 5 ns, '0'
       AFTER 6 ns, '1' AFTER 10 ns, '0' AFTER 12 ns, '1' AFTER 15
       ns, '0' AFTER 16 ns;
  B <= '0', '1' AFTER 2 ns, '0' AFTER 4 ns, '1' AFTER 7 ns, '0'
       AFTER 8 ns, '1' AFTER 9 ns, '0' AFTER 11 ns, '1' AFTER 13 ns,
       '0' AFTER 14 ns;

END ARCHITECTURE;
```

5.4. Test vector

The test vector is a description of the stimuli applied to the system in order to validate its correct operation. Ideally, the test vector has to cover all the possible path in the state diagram. For both versions of the system B, the test vector corresponds to the timing diagram shown in Fig. 5. It is composed of four phases:

- Between 0 and 4, we simulated a pulse on A followed by a pulse on B that do not overlap.
- Between 5 and 8, we simulated a pulse on A followed by a pulse on B that overlaps.
- Between 9 and 12, we simulated a pulse on B followed by a pulse on A that do not overlap.
- Between 13 and 16, we simulated a pulse on B followed by a pulse on A that overlaps.

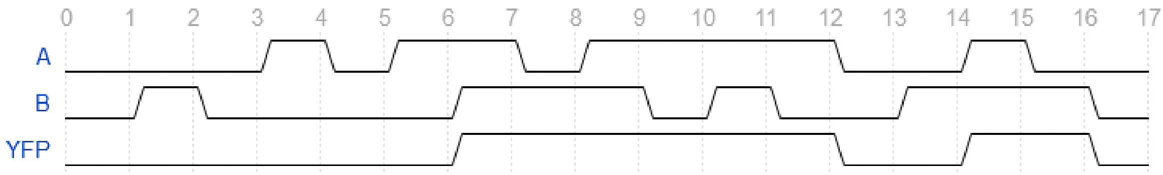

Figure 5. Timing diagram of the test vector and expected response of the system A.

7. Dynamic Model

We established the dynamic models for system B directly from GRN architecture. Only the Gray encoding version has been considered because simulations with the Boolean model already defeated the binary encoding version. The dynamic model is composed of a set of 14 ordinary differential equations (ODEs), one for each input (2), one for each transcription factor (5), one for each associated mRNA (5), one for the reporter (1) and one for the mRNA associated with the reporter (1). Regulations are described as a modulation of the transcription rate according to Hill's equation [5].

Equations (18) to (31) are the dynamic model of the system B. Table 5 summarises the parameters of the models. For computation purpose, the concentrations of all involved molecules are gathered in a state vector. Table 6 gives the way the concentrations of molecules are ordered in this state vector. The Listing 3 is the MATLAB function `SystemB_ODE` which computing the derivative terms of the state vector `dY` as a function of the state vector itself `Y` and the time `t`, *i.e.* equations (18) to (21).

The input stimuli are also encoded in `SystemB_ODE` by computing `betaA` and `betaB` as a function of the time.

$$\frac{d[A]}{dt} = \beta_A(t) - d \cdot [A] \tag{18}$$

$$\frac{d[B]}{dt} = \beta_B(t) - d \cdot [B] \tag{19}$$

$$\begin{aligned}\frac{d[mR1]}{dt} = K_{TR} \cdot \left(\alpha + (1-\alpha) \cdot \frac{[A]^{n_1}}{K_1^{n_1} + [A]^{n_1}} \cdot \frac{K_2^{n_2}}{K_2^{n_2} + [B]^{n_2}}\right) + K_{TR} \\ \cdot \left(\alpha + (1-\alpha) \cdot \frac{K_3^{n_3}}{K_3^{n_3} + [A]^{n_3}} \cdot \frac{[B]^{n_4}}{K_4^{n_4} + [B]^{n_4}}\right) - d_m \cdot [mR1]\end{aligned} \tag{20}$$

$$\frac{d[R1]}{dt} = K_{TL} \cdot [mR1] - d \cdot [R1] \tag{21}$$

$$\frac{d[mR2]}{dt} = K_{TR} \cdot \left(\alpha + (1-\alpha) \cdot \frac{K_5^{n_5}}{K_5^{n_5} + [R1]^{n_5}}\right) - d_m \cdot [mR2] \tag{22}$$

$$\frac{d[R2]}{dt} = K_{TL} \cdot [mR2] - d \cdot [R2] \tag{23}$$

$$\frac{d[mA3]}{dt} = K_{TR} \cdot \left(\alpha + (1-\alpha) \cdot \frac{[X]^{n_{14}}}{K_{14}^{n_{14}} + [X]^{n_{14}}}\right) - d_m \cdot [mA3] \tag{24}$$

$$\frac{d[A3]}{dt} = K_{TL} \cdot [mA3] - d \cdot [A3] \tag{25}$$

$$\frac{d[mX]}{dt} = K_{TR} \cdot \left(\alpha + (1-\alpha) \cdot \frac{K_5^{n_5}}{K_5^{n_5} + [R1]^{n_5}} \cdot \frac{[Y]^{n_6}}{K_6^{n_6} + [Y]^{n_6}}\right) + K_{TR} \cdot \left(\alpha + (1-\alpha) \cdot \frac{K_8^{n_8}}{K_8^{n_8} + [R2]^{n_8}} \cdot \frac{[X]^{n_9}}{K_9^{n_9} + [X]^{n_9}}\right) - d_m \cdot [mX] \tag{26}$$

$$\frac{d[X]}{dt} = K_{TL} \cdot [mX] - d \cdot [X] \tag{27}$$

$$\frac{d[mY]}{dt} = K_{TR} \cdot \left(\alpha + (1-\alpha) \cdot \frac{K_5^{n_5}}{K_5^{n_5} + [R1]^{n_5}} \cdot \frac{[Y]^{n_6}}{K_6^{n_6} + [Y]^{n_6}}\right) + K_{TR} \cdot \left(\alpha + (1-\alpha) \cdot \frac{K_{10}^{n_{10}}}{K_{10}^{n_{10}} + [R2]^{n_{10}}} \cdot \frac{[A3]^{n_{11}}}{K_{11}^{n_{11}} + [A3]^{n_{11}}}\right) - d_m \cdot [mY] \tag{28}$$

$$\frac{d[Y]}{dt} = K_{TL} \cdot [mY] - d \cdot [Y] \tag{29}$$

$$\frac{d[mYFP]}{dt} = K_{TR} \cdot \left(\alpha + (1-\alpha) \cdot \frac{[X]^{n_{12}}}{K_{12}^{n_{12}} + [X]^{n_{12}}} \cdot \frac{[Y]^{n_{13}}}{K_{13}^{n_{13}} + [Y]^{n_{13}}}\right) - d_m \cdot [mYFP] \tag{30}$$

$$\frac{d[YFP]}{dt} = K_{TL} \cdot [mYFP] - d \cdot [YFP] \tag{31}$$

The GRN does not have any specific initialisation mechanism. Thus, at the beginning of the test, when A and B are both low, the system can be either in state S1 or state S5. Simulations of the dynamic model shows that the system always initialises in the S5. This is a consequence of the GRN architecture: without any transcription at the initial state, X and Y are produced. Thus, the MATLAB test vector is a bit different to the one given in Fig 5: an initial pulse of A is added in order to push the system in S1 before the actual start of the simulation.

Listing 3. MATLAB function giving the dynamic model of system B.

```
function dY = SystemB_ODE(t,Y)

     % Nominal value of parameters
     global Ktr alpha K_nom n_nom Ktl dm d

     % Variations
     global sigma_K sigma_n sigma_noise

     % Waveform definition
     global tf

     betaA = 0;
     if (t>1*tf && t<2*tf) || (t>3*tf && t<5*tf) || (t>7*tf && t<8*tf)
      || (t>12*tf && t<14*tf) || (t>17*tf && t<18*tf)
             betaA = 1e-3;
           end

     betaB = 0;
     if (t>4*tf && t<6*tf) || (t>9*tf && t<10*tf) || (t>11*tf &&
         t<13*tf) || (t>15*tf && t<16*tf)
       betaB = 1e-3;
     end

     % Parameter set
     n = max(ones(1,14)*n_nom .* (1+sigma_n*randn(1,14)),0);
     while sum(n>0)<9,
       n = max(ones(1,9)*n_nom .* (1+sigma_n*randn(1,9)),0);
     end
```

```
K = 10.^(log10(ones(1,14)*K_nom) .* (1+sigma_K*randn(1,14)));

% Equations
dY(1)  = betaA - d*Y(1);
dY(2)  = betaB - d*Y(2);
dY(3)  = Ktr*Hill_AR(K(1),n(1),Y(1),K(2),n(2),Y(2)) + ...
         Ktr*Hill_AR(K(4),n(4),Y(2),K(3),n(3),Y(1))-2*dm*Y(3);
dY(4)  = Ktl*Y(3) - d*Y(4);
dY(5)  = Ktr*Hill_R(K(7),n(7),Y(4))- dm*Y(5);
dY(6)  = Ktl*Y(5) - d*Y(6);
dY(7)  = Ktr*Hill_R(K(14),n(14),Y(10))- dm*Y(7);
dY(8)  = Ktl*Y(7) - d*Y(8);
dY(9)  = Ktr*Hill_AR(K(9),n(9),Y(10),K(8),n(8),Y(6)) + ...
         Ktr*Hill_AR(K(6),n(6),Y(12),K(5),n(5),Y(4))-2*dm*Y(9);
dY(10) = Ktl*Y(9) - d*Y(10);
dY(11) = Ktr*Hill_AR(K(11),n(11),Y(8),K(10),n(10),Y(6)) + ...
         Ktr*Hill_AR(K(6),n(6),Y(12),K(5),n(5),Y(4))- 2*dm*Y(11);
dY(12) = Ktl*Y(11) - d*Y(12);
dY(13) = Ktr*Hill_AA(K(12),n(12),Y(10),K(13),n(13),Y(12))-dm*Y(13);
dY(14) = Ktl*Y(13) - d*Y(14);

dY = dY' + randn(14,1)*sigma_noise;
```

Table 5. List of specific parameters.

Symbol	Description
K_1	Dissociation constant of A on the promoter of the operon #1
K_2	Dissociation constant of B on the promoter of the operon #1
K_3	Dissociation constant of A on the promoter of the operon #2
K_4	Dissociation constant of B on the promoter of the operon #2
K_5	Dissociation constant of R1 on the promoter of the operon #3
K_6	Dissociation constant of Y on the promoter of the operon #3
K_7	Dissociation constant of R1 on the promoter of the operon #4
K_8	Dissociation constant of R3 on the promoter of the operon #5
K_9	Dissociation constant of X on the promoter of the operon #5
K_{10}	Dissociation constant of R3 on the promoter of the operon #6
K_{11}	Dissociation constant of A4 on the promoter of the operon #6
K_{12}	Dissociation constant of X on the promoter of the operon #7
K_{13}	Dissociation constant of Y on the promoter of the operon #7
K_{14}	Dissociation constant of X on the promoter of the operon #8
n_1	Hill's number for the repression of the promoter of the operon #1 by A
n_2	Hill's number for the repression of the promoter of the operon #1 by B
n_3	Hill's number for the activation of the promoter of the operon #2 by A
n_4	Hill's number for the repression of the promoter of the operon #2 by B
n_5	Hill's number for the repression of the promoter of the operon #3 by R1
n_6	Hill's number for the repression of the promoter of the operon #3 by Y
n_7	Hill's number for the repression of the promoter of the operon #4 by R1
n_8	Hill's number for the repression of the promoter of the operon #5 by R2
n_9	Hill's number for the repression of the promoter of the operon #5 by X
n_{10}	Hill's number for the repression of the promoter of the operon #6 by R2
n_{11}	Hill's number for the repression of the promoter of the operon #6 by A3
n_{12}	Hill's number for the repression of the promoter of the operon #7 by X
n_{13}	Hill's number for the repression of the promoter of the operon #7 by Y
n_{14}	Hill's number for the repression of the promoter of the operon #8 by X

Table 6. Composition of the state vector.

Index	Description	Description
1	$[A]$	Concentration of A
2	$[B]$	Concentration of B
3	$[mR1]$	Concentration of mRNA for R1
4	$[R1]$	Concentration of R1
5	$[mR2]$	Concentration of mRNA for R2
6	$[R2]$	Concentration of R2
7	$[mA3]$	Concentration of mRNA for A3
8	$[A3]$	Concentration of A3
9	$[mX]$	Concentration of mRNA for X
10	$[X]$	Concentration of X
11	$[mY]$	Concentration of mRNA for Y
12	$[Y]$	Concentration of Y
13	$[mYFP]$	Concentration of mRNA for YFP
14	$[YFP]$	Concentration of YFP

8. Open-loop GRN

The open-loop GRN is obtained by deleting the repression of the operon #4 by X and adding a new input X_o that represses the operon #4 directly. The sketch of this new GRN is shown in Fig. 6. The timing diagram used for the simulation of the open-loop GRN is given in Fig. 7.

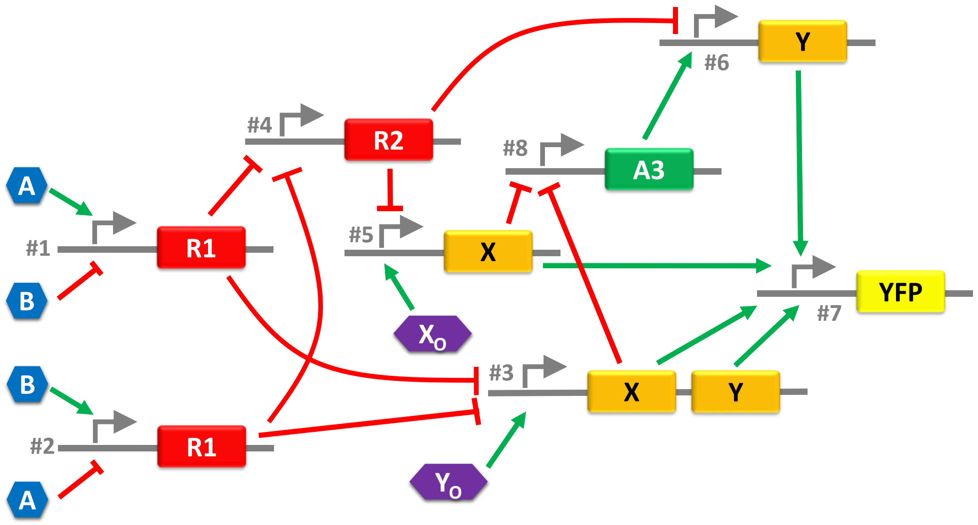

Figure 6. Sketch of the open-loop GRN. This GRN is now composed of three inputs and one output. Its associated Boolean function is equation (1) and (2)

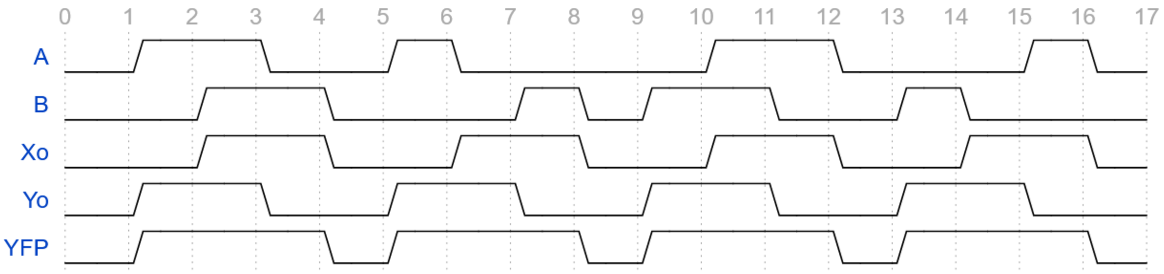

Figure 7. Timing diagram of the test vector and expected response of the system B in an open loop.

9. References

1. Micheli GD. Synthesis and optimization of digital circuits. McGraw-Hill Higher Education; 1994.

2. Madec M, Pecheux F, Gendrault Y, Rosati E, Lallement C, Haiech J. GeNeDA: An Open-Source Workflow for Design Automation of Gene Regulatory Networks Inspired from Microelectronics. Journal of Computational Biology. 2016;23. doi:10.1089/cmb.2015.0229

3. Shin J, Zhang S, Der BS, Nielsen AA, Voigt CA. Programming Escherichia coli to function as a digital display. Molecular Systems Biology. 2020;16: e9401. doi:10.15252/msb.20199401

4. Ashenden PJ. The Designer's Guide to VHDL. Morgan Kaufmann; 2010.

5. Konkoli Z. Safe uses of Hill's model: an exact comparison with the Adair-Klotz model. Theoretical biology & medical modelling. 2011;8: 10–10. doi:10.1186/1742-4682-8-10.
