# Supplementary material for: Feasibility and reliability of sequential logic with gene regulatory networks
Source: PLoS One. 2021 Mar 30;16(3):e0249234. doi: 10.1371/journal.pone.0249234 (PMC8009411; doi:10.1371/journal.pone.0249234)
Supplement: S3 File — This file contains examples of parameters distribution and a table with the range of parameters as a function of the inhomogeneity value for Hill’s number and dissociation constant. (PDF) [file pone.0249234.s003.pdf]

Feasibility and reliability of sequential logic with gene regulatory networks

Morgan Madec[1]*, Elise Rosati[1], Christophe Lallement[1]

[1]Laboratory of Engineering Sciences, Computer Sciences and Imaging, UMR 7357 (University of Strasbourg / CNRS), 300 boulevard Sebastien Brandt, 67412 Illkirch, France. *Corresponding author, e-mail: morgan.madec@unistra.fr

Supporting Information 3

Impact of the Parameters Inhomogeneity

The protocol we applied to assess the impact of the parameters inhomogeneity on the operation of a given sequential system is the following:

- First, we define the set of nominal parameters for the dynamic model of the system.
- Second, the focus is put on two specific parameters: the effective dissociation constant between the promoter and the transcription factor (K_A or K_R depending on whether the transcription factor is an activator or a repressor) and the Hill's number (n_A or n_R depending on whether the transcription factor is an activator or a repressor). We let these parameters change from one regulation to the other whereas the other parameters will be at the same value whatever the regulation. The set of K_A, K_R, n_A and n_R for each regulation compose what will be called in the following the set of regulation parameters.
- Third, for a given system and for a given inhomogeneity parameter σ, 100 sets of regulation parameters are randomly generated. The way they are generated is the topic of this Supporting Information.
- Fourth, the dynamic system is run with each set of regulation parameters and the simulation results is compared to the expected behavior.

The metric for this evaluation is the percentage of successful simulations for a given value of σ.

1. Set of Nominal Parameters and Set of Regulation Parameters

The set of nominal parameters is given in Table 1. All these parameters except K_A, K_R, n_A and n_R are the same for each regulation of each system. For K_A, K_R, n_A and n_R, the value given in the Table is the nominal value called respectively K_0 for K_A, K_R and n_0 for n_A, n in the following.

The set of regulation parameters for systems A and B are respectively given in Table 2 and Table 3.

Table 1. Set of nominal parameters for both systems.

$k_{TR,eff}$	Maximal effective transcription rate (to obtain normalized conc.)	0.002 s^{-1}
α	Promoter leakiness	0.001
K_A	Dissociation constant of the activator with its binding site	0.03
K_R	Dissociation constant of the repressor with its binding site	0.03
n_A	Hill's number for the activator	2
n_R	Hill's number for the repressor	2
$k_{TL,eff}$	Effective translation rate (to obtain normalized concentrations)	10^{-3} s^{-1}
d_{mRNA_i}	Degradation rate for the mRNA encoding for the protein P_i	0.002 s^{-1}
$d_{eff,i}$	Degradation rate for the protein P_i	10^{-3} s^{-1}

Table 2. Set of regulation parameters for the system A.

Symbol	Description
K_1	Dissociation constant of B on the promoter of the operon #1
K_2	Dissociation constant of R1 on the promoter of the operon #2
K_3	Dissociation constant of A on the promoter of the operon #2
K_4	Dissociation constant of A on the promoter of the operon #3
K_5	Dissociation constant of B on the promoter of the operon #3
K_6	Dissociation constant of R2 on the promoter of the operon #4
K_7	Dissociation constant of X on the promoter of the operon #4
K_8	Dissociation constant of R3 on the promoter of the operon #5
K_9	Dissociation constant of R4 on the promoter of the operon #5
n_1	Hill’s number for the repression of the promoter of the operon #1 by B
n_2	Hill’s number for the repression of the promoter of the operon #2 by R1
n_3	Hill’s number for the activation of the promoter of the operon #2 by A
n_4	Hill’s number for the repression of the promoter of the operon #3 by A
n_5	Hill’s number for the repression of the promoter of the operon #3 by B
n_6	Hill’s number for the repression of the promoter of the operon #4 by R2
n_7	Hill’s number for the repression of the promoter of the operon #4 by X
n_8	Hill’s number for the repression of the promoter of the operon #5 by R3
n_9	Hill’s number for the repression of the promoter of the operon #5 by R4

2. Hill’s number

For the p-th run, the q-th Hill’s number $n_q^{(p)}$ is computed as following:

$$n_q^{(p)} = n_0 \cdot \Psi(1, \sigma \cdot n_0) \quad (1)$$

where $n_0 = 2$ is the nominal value for the Hill’s number and $\Psi(1, \sigma)$ is a randomly drawn value from a Gaussian distribution centered on 1 and with a standard deviation of $\sigma \cdot n_0$. The parameter σ is called the inhomogeneity parameter. It should be noticed that negative values of $n_q^{(p)}$ does not make sense from a biological perspective. A test is performed to exclude parameters sets that contains at least one negative $n_q^{(p)}$.

The distribution of $n_q^{(p)}$ for different values of σ are given in Fig. 1. Additionally, the Table 4 gives an overview of the range of value for $n_q^{(p)}$ as a function of σ.

Table 3. Set of regulation parameters for the System B.

Symbol	Description
K_1	Dissociation constant of A on the promoter of the operon #1
K_2	Dissociation constant of B on the promoter of the operon #1
K_3	Dissociation constant of A on the promoter of the operon #2
K_4	Dissociation constant of B on the promoter of the operon #2
K_5	Dissociation constant of R1 on the promoter of the operon #3
K_6	Dissociation constant of Y on the promoter of the operon #3
K_7	Dissociation constant of R1 on the promoter of the operon #4
K_8	Dissociation constant of R3 on the promoter of the operon #5
K_9	Dissociation constant of X on the promoter of the operon #5
K_{10}	Dissociation constant of R3 on the promoter of the operon #6
K_{11}	Dissociation constant of A4 on the promoter of the operon #6
K_{12}	Dissociation constant of X on the promoter of the operon #7
K_{13}	Dissociation constant of Y on the promoter of the operon #7
K_{14}	Dissociation constant of X on the promoter of the operon #8
n_1	Hill's number for the repression of the promoter of the operon #1 by A
n_2	Hill's number for the repression of the promoter of the operon #1 by B
n_3	Hill's number for the activation of the promoter of the operon #2 by A
n_4	Hill's number for the repression of the promoter of the operon #2 by B
n_5	Hill's number for the repression of the promoter of the operon #3 by R1
n_6	Hill's number for the repression of the promoter of the operon #3 by Y
n_7	Hill's number for the repression of the promoter of the operon #4 by R1
n_8	Hill's number for the repression of the promoter of the operon #5 by R2
n_9	Hill's number for the repression of the promoter of the operon #5 by X
n_{10}	Hill's number for the repression of the promoter of the operon #6 by R2
n_{11}	Hill's number for the repression of the promoter of the operon #6 by A3
n_{12}	Hill's number for the repression of the promoter of the operon #7 by X
n_{13}	Hill's number for the repression of the promoter of the operon #7 by Y
n_{14}	Hill's number for the repression of the promoter of the operon #8 by X

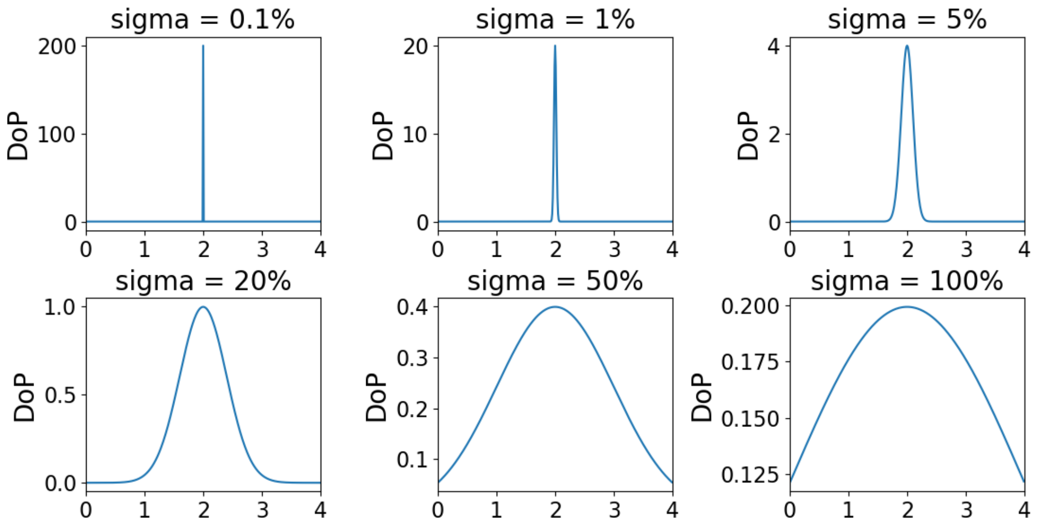

Figure 1. Distribution of Hill's number as a function of the inhomogeneity parameter σ. DoP stands for "distribution of probabilities".

Table 4. Range of values for Hill's number as a function of the inhomogeneity parameter σ. Only 1 value over 15787 is outside this range.

Dispersion parameter	Range in which 99.99 % of the drawn values are
$\sigma = 0.1\%$	$[1.992\,;2.008]$
$\sigma = 1\%$	$[1.92\,;2.08]$
$\sigma = 5\%$	$[1.6\,;2.4]$
$\sigma = 20\%$	$[0.4\,;3.6]$
$\sigma = 50\%$	$]0;6]$
$\sigma = 100\%$	$]0;10]$

3. Dissociation constant

For the p-th run, the q-th dissociation constant $K_q^{(p)}$ is computed as following:

$$K_q^{(p)} = K_0 \cdot 10^{\Psi(0,\sigma\cdot\log_{10} K_0)} \quad (2)$$

where $K_0 = 0.03$ is the nominal value for the Hill's number and $\Psi(0, \sigma \cdot \log_{10} K_0)$ is a randomly drawn value from a Gaussian distribution centered on 0 and with a standard deviation of $\sigma \cdot \log_{10} K_0$. The parameter σ is called the inhomogeneity parameter.

The distribution of $K_q^{(p)}$ for different values of σ are given in Fig. 2. Additionally, the Table 5 gives an overview of the range of value for $K_q^{(p)}$ as a function of σ.

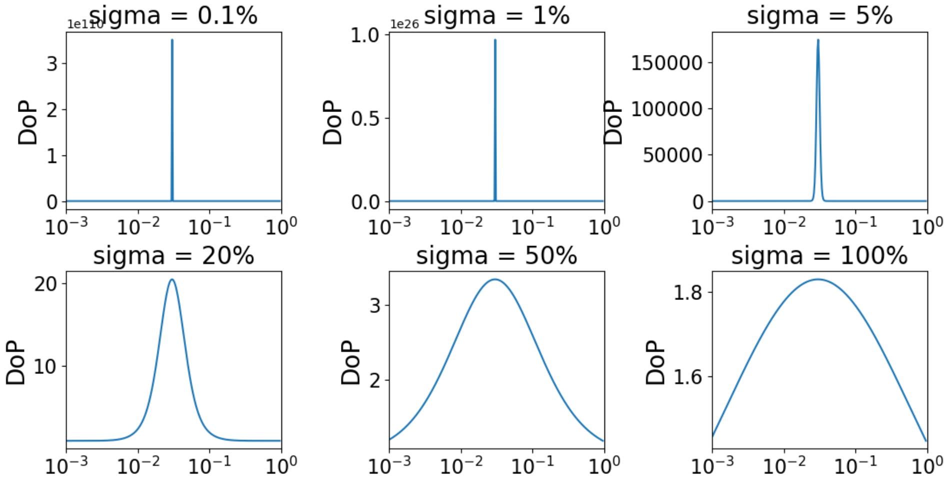

Figure 2. Distribution of dissociation constant as a function of the inhomogeneity parameter σ. DoP stands for "distribution of probabilities".

Table 5. Range of values for the dissociation constant as a function of the inhomogeneity parameter σ. Only 1 value over 15787 is outside this range.

Dispersion parameter	Range in which 99.99 % of the drawn values are
$\sigma = 0.1\%$	$[0.0295\,;0.0306]$
$\sigma = 1\%$	$[0.0250\,;0.0361]$
$\sigma = 5\%$	$[0.0120\,;0.0755]$
$\sigma = 20\%$	$[0.0007\,;1.1967]$
$\sigma = 50\%$	$[3\cdot 10^{-6}\,;300.60]$
$\sigma = 100\%$	$[3\cdot 10^{-10}\,;3\cdot 10^{6}]$
