# Supplementary material for: Feasibility and reliability of sequential logic with gene regulatory networks
Source: PLoS One. 2021 Mar 30;16(3):e0249234. doi: 10.1371/journal.pone.0249234 (PMC8009411; doi:10.1371/journal.pone.0249234)
Supplement: S4 File — This file details an example of failing simulation for the system A. (PDF) [file pone.0249234.s004.pdf]

Feasibility and reliability of sequential logic with gene regulatory networks

Morgan Madec[1]*, Elise Rosati[1], Christophe Lallement[1]

[1]Laboratory of Engineering Sciences, Computer Sciences and Imaging, UMR 7357 (University of Strasbourg / CNRS), 300 boulevard Sebastien Brandt, 67412 Illkirch, France. *Corresponding author, e-mail: morgan.madec@unistra.fr

Supporting Information 4

Example of failing simulations

This file provides an example of failing simulations. It occurs with the system A during a transition from state S1 to S2 (the state diagram of the system is reminded in Fig 1) induced by the rising of the input B. The set of regulation parameters (Table 1) is obtained with an inhomogeneity parameter of 20 %. According to the results given in Figure 6 of the main manuscript, with such inhomogeneity, the system has about 50% of chance to fail.

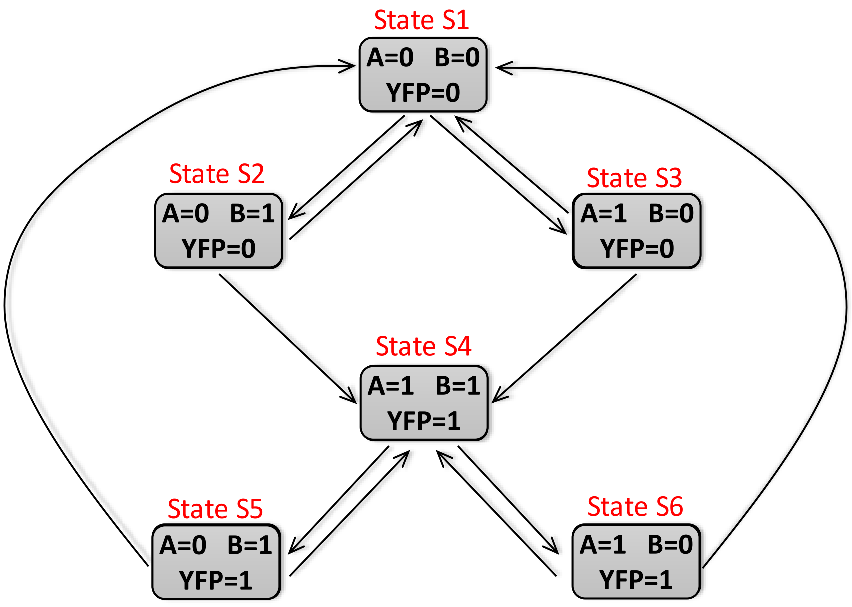

Figure 1. State diagram of system A. The system is composed of six states.

Let us dissect this failing transition. At $t = 100$, the system is in state S1 and B rises to 1. The expected behavior is a transition to state S2. According to transition Karnaugh map of the system (Table 3 in Supporting Information 1), despite the rising of B, the internal variable X as to stay low. According to sketch of the GRN (reminded in Fig 2), the consequences of the rising edge of B are:

- $R1$ is expected to fall from 1 to 0
- $R2$ is expected to stay at 0 because A stay at 0.
- $R3$ is expected to fall from 1 to 0
- $R4$ is expected to stay high because no repressor is expressed.
- X is repressed to stay low because one of its repressors, namely $R4$, stay high.

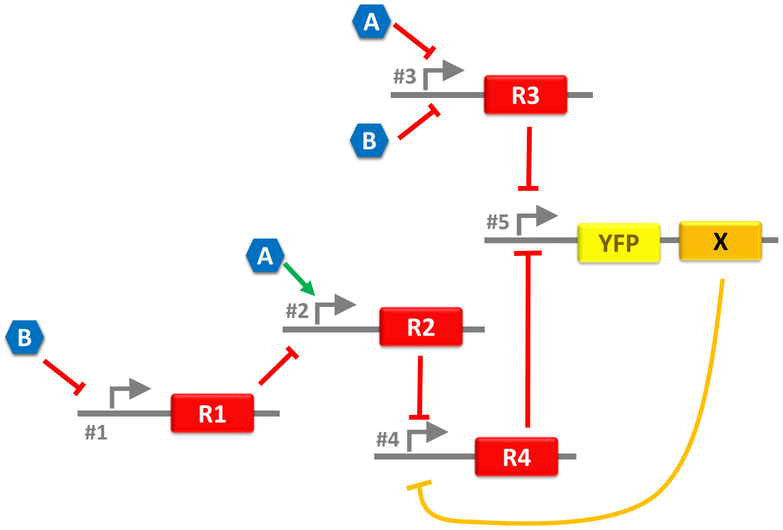

Figure 2. GRN inferred for the system A. The GRN is composed of 5 operons and 5 transcription factors.

Table 1. List of inhomogeneity parameters.

Symbol	Description	Value
K_1	Dissociation constant of B on the promoter of the operon #1	0.0256
K_2	Dissociation constant of R1 on the promoter of the operon #2	0.0493
K_3	Dissociation constant of A on the promoter of the operon #2	0.0535
K_4	Dissociation constant of A on the promoter of the operon #3	0.0323
K_5	Dissociation constant of B on the promoter of the operon #3	0.0347
K_6	Dissociation constant of R2 on the promoter of the operon #4	0.1106
K_7	Dissociation constant of X on the promoter of the operon #4	0.0121
K_8	Dissociation constant of R3 on the promoter of the operon #5	0.0137
K_9	Dissociation constant of R4 on the promoter of the operon #5	0.0256
n_1	Hill's number for the repression of the promoter of the operon #1 by B	2.298
n_2	Hill's number for the repression of the promoter of the operon #2 by R1	1.374
n_3	Hill's number for the activation of the promoter of the operon #2 by A	1.753
n_4	Hill's number for the repression of the promoter of the operon #3 by A	1.981
n_5	Hill's number for the repression of the promoter of the operon #3 by B	1.870
n_6	Hill's number for the repression of the promoter of the operon #4 by R2	1.758
n_7	Hill's number for the repression of the promoter of the operon #4 by X	1.701
n_8	Hill's number for the repression of the promoter of the operon #5 by R3	1.408
n_9	Hill's number for the repression of the promoter of the operon #5 by R4	2.663

For a good interpretation of the simulation results, it should be reminded that the lower the dissociation constant, the stronger the regulation. Simulation shows that during the transition, the repression of X by $R3$ is released but R4 is still produced, which is compliant with the expectations. However, the repression of $R3$ is ten times stronger compared to the repression of $R4$ ($K_9 \simeq 10 \cdot K_8$). $R4$ alone is not strong enough to maintain a complete inhibition of X. Thus, the concentration of X increase a little bit. In the meantime, the repression of $R4$ by X is also very strong. As a consequence, a small quantity of X is enough to decrease the production of $R4$ which, in turn, tends to increase again the concentration of X. Ultimately, an unexpected transition of X from 0 to 1 occurs. The system thus goes to state S5 rather than to state S2. Once this bad transition had occurred, it is impossible for the system to return to normal operation, except by chance due to another bad transition. In Figure 3, the light decrease of $R4$ and increase of X can be observed between $t = 102$ and $t = 104$, then intensifies after $t = 104$.

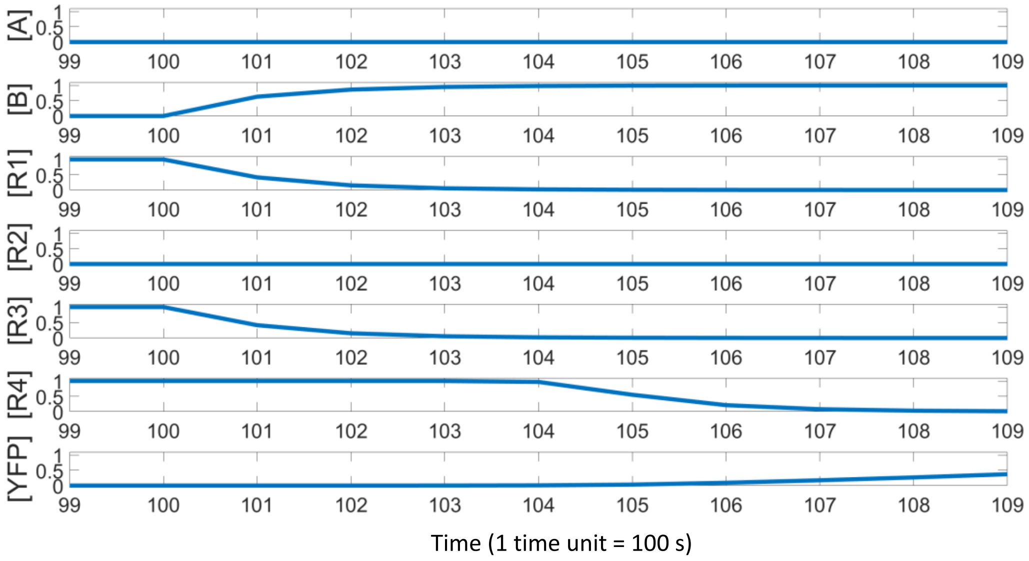

Figure 3. Simulation results of the system A with the parameters given in Table 1. The figure is a zoom around the transition that induces the failure. One time unit on the x-axis corresponds to 100 seconds.
