# Supplementary figures and images for: Feasibility and reliability of sequential logic with gene regulatory networks
Source: PLoS One. 2021 Mar 30;16(3):e0249234. doi: 10.1371/journal.pone.0249234 (PMC8009411; doi:10.1371/journal.pone.0249234)

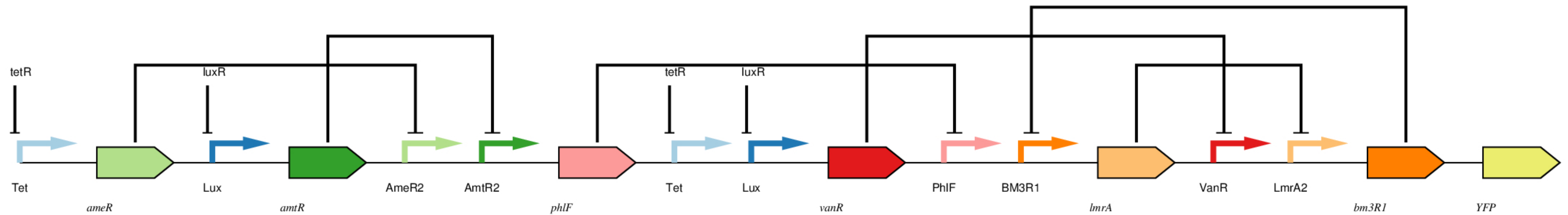

Supplement: S5 File — (PDF) [file pone.0249234.s005.pdf]

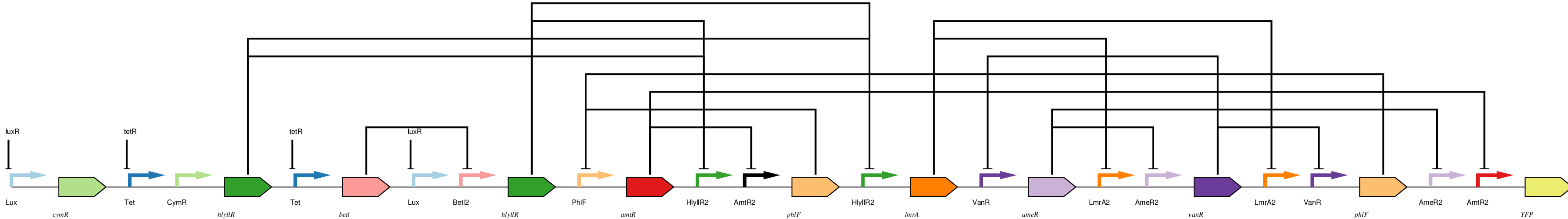

Supplement: S6 File — (PDF) [file pone.0249234.s006.pdf]
